# Azithromycin in severe malaria bacterial co-infection in African children (TABS-PKPD): a phase II randomised controlled trial

**DOI:** 10.1186/s12916-024-03712-5

**Published:** 2024-11-06

**Authors:** Roisin Connon, Peter Olupot-Olupot, Arthur M. A. Pistorius, William Okiror, Tonny Ssenyondo, Rita Muhindo, Sophie Uyoga, Ayub Mpoya, Thomas N. Williams, Diana M. Gibb, A. Sarah Walker, Rob ter Heine, Elizabeth C. George, Kathryn Maitland

**Affiliations:** 1 MRC Clinical Trials Unit at University College London, Aviation House, 125 Kingsway, London, WC28 6NH UK; 2 Mbale Clinical Research Institute, Pallisa Road, Mbale, PO Box 291, Uganda; 3 Faculty of Health Sciences, Busitema University, Mbale Regional Referral Hospital, Mbale, Uganda; 4 Department of Pharmacy, Research Institute for Medical Innovation, Radboudumc, Nijmegen, The Netherlands; 5 KEMRI-Wellcome Trust Research Programme, Kilifi, PO Box 230, Kenya; 6 Department of Infectious Disease and Institute of Global Health and Innovation, Division of Medicine, Imperial College, London, W2 1PG UK

**Keywords:** Severe malaria, African children, Bacterial infection, Pharmacokinetics, Clinical trial

## Abstract

**Background:**

African children with severe malaria are at increased risk of non-typhoidal salmonellae co-infection. Broad-spectrum antibiotics are recommended by guidelines but the optimal class and dose have not been established. We investigated the optimal dose of oral dispersible azithromycin and whether simple clinical criteria and point-of-care biomarkers could target antibiotics to those at greatest risk of bacterial co-infection.

**Methods:**

We conducted a phase I/II trial in Ugandan children with severe malaria comparing a 5-day course of azithromycin: 10, 15 and 20 mg/kg of azithromycin (prescribed by weight bands) spanning the dose-range effective for other salmonellae infection. We generated relevant pharmacokinetic (PK) data by sparse sampling during dosing intervals and investigated associations between azithromycin exposure and potential mechanisms (PK-pharmacodynamics) using change in C-reactive protein (CRP), a putative marker of sepsis, at 72 h (continuous) and microbiological cure (7-day) (binary), alone and as a composite with 7-day and 90-day survival. To assess whether clinical or biomarkers could identify those at risk of sepsis, a non-severe malaria control was concurrently enrolled.

**Results:**

Between January 2020 and January 2022, 105 cases were randomised azithromycin doses: 35 to 10 mg/kg, 35 to 15 mg/kg and 35 to 20 mg/kg. Fifty non-severe malaria controls were concurrently enrolled. CRP reduced in all arms by 72 h with a mean reduction of 65.8 mg/L (95% CI 57.1, 74.5) in the 10 mg/kg arm, 64.8 mg/L (95% CI 56.5, 73.1; *p* = 0.87) in the 20 mg/kg arm and a smaller reduction 51.2 mg/L (95% CI 42.9, 59.5; *p* = 0.02) in the 15 mg/kg arm. Microbiological cure alone outcome was not analysed as only one pathogen was found among cases. Three events contributed to the composite outcome of 7-day survival and microbiological cure, with no events in the 15 mg/kg arm. The odds ratio comparing 20 vs 10 mg/kg was 0.50 (95% CI 0.04, 5.79); *p* = 0.58. Due to the low number of pathogens identified, it was not possible to identify better methods for targeting antibiotics including both the cases and controls.

**Conclusions:**

We found no evidence for an association between systemic azithromycin exposure and reduction in CRP. Further work is needed to better identify children at highest risk from bacterial co-infection.

**Trial registration:**

ISRCTN49726849 (registered on 27th October 2017).

**Supplementary Information:**

The online version contains supplementary material available at 10.1186/s12916-024-03712-5.

## Background

Severe *Plasmodium falciparum* malaria remains a common cause of paediatric hospital admission in a large part of sub-Saharan Africa. In 2021, the World Health Organization (WHO) estimated that there were ~ 2000 African childhood deaths each day due to severe *P. falciparum* malaria [1]. The definitive treatment for severe malaria is parenteral artesunate; however, in-hospital mortalities of 7–10% are still common [2]. The other antimicrobial therapy recommended by guidelines for all children with severe malaria is parenteral broad-spectrum antibiotics [3]. These recommendations are based on two reasons. First, 4–16% of children with severe malaria have bacterial co-infection, which is largely due to enteric Gram-negative organisms with a predominance of non-typhoidal salmonella (NTS) species [4]. Children with co-infection have substantially higher case fatality, estimated at 24% compared to 10% in children without co-infection [4]. In 2011, it was estimated one third of all severe malaria deaths in Kenyan children were attributable to bacterial co-infection [5]. Second, the clinical presentation of severe malaria and severe sepsis (of bacterial aetiology) are often indistinguishable [6]. Thus, for children with a positive rapid diagnostic test (often the only test which is carried out to diagnose malaria rather than thick film microscopy) who have a low burden of malaria parasites, the clinical presentation is often caused by sepsis rather than severe malaria [6]. It has been shown that invasive bacterial infection is more common in these children compared to those with higher parasite burdens, estimated by *P. falciparum* histidine-rich protein 2 (*Pf*HRP2) levels [7].

WHO definitions of severe malaria are very broad and thus apply to a large proportion of paediatric admissions in endemic regions. Overall, case fatality is relatively low (1–2%) [8] and thus, those meeting these definitions are unlikely to benefit from antimicrobial therapy as a whole. Refinements to the criteria for severe malaria to increase specificity, incorporating platelet counts < 150,000 per µL and *Pf*HRP2 > 1000 ng/mL, have been proposed for epidemiological studies and clinical trials [9]. However, these are based on mortality outcomes and cannot be used to define a particular population at risk of bacterial infection. A prospective study conducted in Teule, Tanzania, examining clinical and laboratory features of children with *P. falciparum* malaria (defined by either positive blood film or a positive Paracheck™ (*Pf*HRP2) rapid diagnostic test for malaria (RDT)) found that an axillary temperature > 38 °C or < 36 °C with one or more of impaired consciousness (prostration or unconsciousness), respiratory distress, Hb < 5 g/dL or a positive HIV test identified 85% of bacterial co-infections [7]. Children with these criteria had a threefold higher mortality than children admitted with malaria without these criteria. These criteria were considered to be a good starting point for eligibility for evaluating antibiotic strategies in severe malaria since they could enrich any study population with those at greatest risk.

With respect to antimicrobial treatment, the choice of antibiotics should be active against NTS, the commonest cause of bacterial co-infection, requiring intracellular penetration, and also be active against other Gram-negative infections. Some of the commonly recommended antibiotics antimicrobials (e.g. ampicillin/gentamicin combination) do not possess this property and are thus ineffective. For NTS specifically, the efficacy of gentamicin is doubtful and susceptibility testing unreliable due to the pathogen’s intracellular nature [10, 11]. Third-generation cephalosporins (e.g. ceftriaxone) are the most widely used antimicrobials but resistance is becoming widespread [12, 13]. Also, if given cephalosporins few children will receive an ‘adequate’ therapeutic dose, since NTS requires ~ 7 days parenteral therapy thus requiring prolonged hospital stay (median 4–5 days) which may be interrupted early due to economic barriers.

There are several reasons why we considered that azithromycin represented an attractive option for adjunctive antimicrobial therapy in severe malaria. Given the worldwide threat of antimicrobial resistance, the underlying principle is to test the narrowest spectrum antibiotic that would be practical, generalisable and plausibly have reasonable efficacy. Primarily, azithromycin is active against NTS as well as a range of other Gram-negative and Gram-positive organisms. It is licenced for use and has a good safety profile in children. Azithromycin’s pharmacokinetic properties include extensive tissue distribution, prolonged phagocyte concentrations and a longer elimination half-life (~ 50 h) than other macrolides, thus enabling once-daily dosing (versus more frequent dosing required for clindamycin—a lincosamide antibiotic with similar mode of actions as macrolides) [14]. Macrolides also have beneficial properties independent of their antimicrobial effects [15] on immune activation. Moreover, randomised controlled trials have shown that orally prescribed azithromycin is an effective treatment for enteric fever [16, 17].

Children generally tolerate a wide range of azithromycin doses (5–20 mg/kg). However, the evidence supporting efficacy and optimal dose for azithromycin treatment of co-infection in severe malaria is lacking. We therefore conducted a clinical study with two major aims. First, to establish the optimal dose of oral dispersible azithromycin as an antimicrobial treatment for children with severe malaria via population pharmacokinetic (PK) modelling and a phase II clinical trial. Rather than calculate azithromycin doses based on body weight (which in practice is poorly implemented), the trial used weight bands. The doses of 10, 15 and 20 mg/kg for the trial were developed based on existing data [18] and span the lowest to highest mg/kg doses demonstrated to be equally effective as parenteral treatment for other salmonellae infection. The nested PK sub-study aimed to validate the results from the randomised trial. Second, many hospitals in Africa lack quality controlled culture facilities and even when quality-controlled blood-culture facilities are available, low sensitivity (due to frequent pre-hospital antibiotic therapy, low culture volumes from children and low bacterial density) and long times to culture-positivity (typically 2–3 days) mean that alternative approaches for identifying concurrent Gram-negative bacteraemia would be highly valuable. We therefore aimed to investigate whether a combination of clinical, point-of-care diagnostic tests and/or biomarkers can accurately identify the sub-group of severe malaria with culture-proven bacteraemia and enable antimicrobials to be targeted to those at greatest risk by analysing data from children enrolled in the trial and a contemporaneous control cohort of children who were hospitalised with non-severe malaria (not meeting Teule criteria).

## Methods

### Study design

The study included of two cohorts: children with severe malaria defined by the Teule criteria (‘cases’) and those with malaria not meeting the criteria (‘controls’). Among the cases, we conducted an open-label phase I/II randomised trial in order to compare three doses of azithromycin in children with severe malaria at high risk of bacterial infection (TABS trial). Eligible children were randomised 1:1:1 to 10, 15 or 20 mg/kg taken orally over 5 days. The control cohort were not included in this randomisation, but enrolled separately and treated with standard of care. The protocol for this study has been previously published [19]. Trial registration ISRCTN**49726849** (registered on 27th October 2017).

### Study population

The inclusion criteria applying to both cases and controls were aged 6 months to 12 years with axillary temperature > 38 °C or < 36 °C, with evidence of malaria from a positive blood film or ParaCheck Pf® rapid diagnostic test malaria (RDT) and parents willing to provide consent. Additionally in order to be enrolled as a case, the child had to meet any one of the following criteria: impaired consciousness (prostration or unconsciousness); respiratory distress; severe anaemia (haemoglobin < 5 g/dL) or HIV infection (the Teule criteria: as group identified as high risk for bacterial infection in a study by Nadjm et al. in Tanzania) [7]. Exclusion criteria applying to cases only were major contraindications to azithromycin and concomitant use of interacting drugs. The study took place in Mbale Regional Referral Hospital in Eastern Uganda, an area of high malaria endemicity.

### Study medication

Azithromycin was donated for the trial by Cipla Limited (Cipla House, Peninsula Business Park, Ganpatrao Kadam Marg, Lower Parel, Mumbai, 400 013, Maharashtra, India). Cases were prescribed once daily doses of azithromycin over 5 days. Rather than calculate azithromycin doses based on body weight, the trial used weight band dosing (see Supplementary Table 1) all using 100 mg dispersible tablets. The individual doses were rounded to the nearest 50 mg to facilitate dosing in practice, based on tablet sizes of 100 mg. As the trial was designed to target the use of ‘appropriate’ antimicrobial treatments, from admission to the trial up to 72 h clinicians were permitted to also prescribe antibiotics (excluding cephalosporins, quinolones or macrolides) based on the child’s clinical condition and according to country guidelines. On day 3, i.e. 72 h later (or earlier if microbiological and susceptibility results were available), clinicians were permitted to prescribe broad-spectrum antimicrobials guided by the admission blood culture results. Since the control group were at low risk of bacterial co-infection, they did not routinely receive antibiotics as per national and WHO guidelines.

### Outcome measures for randomised comparison

The co-primary outcome measures for the randomised comparison were change in C-reactive protein (CRP) from baseline to 72 h and microbiological cure at 7 days, alone and as a composite with 7-day survival. Secondary efficacy outcomes included mortality at 48 h, day 28 and day 90; length of hospital stay and hospital readmission by 90 days. Secondary safety outcomes were serious adverse events (SAEs), grade 3/4 adverse events (AEs), AEs that were definitely, probably or possibly related to azithromycin, and AEs leading to a change in azithromycin.

### Sample size

The trial aimed to generate pilot efficacy data on the optimal azithromycin dose for children with severe malaria to inform the design of a later phase III trial and so a formal sample size was not calculated. The numbers required to address the trial objectives were therefore balanced against the exposure of children in these settings to a therapeutic intervention (dose) for which there are limited data to date.

One hundred five children were considered sufficient for the PKPD sampling and modelling to determine an optimal dose in children with severe malaria using change in CRP at 72 h. This was under the assumption that 20% of enrolled children (meeting the severity criteria and receiving azithromycin) would have bacteraemia and that 80% of these infections would be caused by non-typhoidal salmonellae or other enteric Gram-negative organisms based on previous research.

There was no formal sample size for the control cohort. The target was selected to be achievable by the study site in the same time span as the case cohort.

### Screening and randomisation

Potentially eligible children were identified by study nurses or clinicians, assessed clinically and given a rapid bedside malaria test (ParaCheck Pf®) to determine malaria status. Written informed consent from parents/guardians was required before enrolment for controls and cases who were in a stable condition. A deferred consent procedure was approved for cases who were admitted in an emergency, where the full procedure would have caused an unacceptable delay in treatment. For these cases, verbal assent was sought prior to enrolment, with full written consent obtained once the child’s condition was stabilised.

The randomisation list was computer generated by a statistician at the MRC CTU using permuted blocks. Sealed opaque envelopes containing the trial number and treatment allocation, visible only once opened, were prepared at the Clinical Trials Facility in KWTP in Kilifi. The envelopes were numbered consecutively and opened in strict numerical order by the study doctor or the study nurse at the site at the time of randomisation, for children eligible to be enrolled as a case.

Children enrolled as controls were not subject to the randomisation procedure. These children were assigned a study ID number following consent.

### Clinical monitoring and study assessments

Following randomisation, azithromycin was prescribed using weight band-based dosing and administered once daily by study nurses. Dispersible azithromycin tablets were given orally, and via a nasogastric tube for those not able to take orally. The dose was repeated if patient vomited within 30 min of dose administration. In both cases and controls, all study patients received standard of care including antimalarial drugs—parenteral artesunate given over 3 days and followed by an oral course of Coartem (artemether/lumefantrine) following national guidelines. Other management (e.g. transfusion, intravenous fluids and antipyretics prescriptions) was based on WHO syndromic patient management. Children discharged before day 5 were provided with a supply of azithromycin tablets to complete their course. Controls received usual standard of care, including antibiotics only if clinically indicated after enrolment. Clinical assessments were carried by study nurses or doctors out at 30 min, 60 min, 90 min, 2, 4, 8, 24, 36 and 48 h from randomisation/enrolment while in hospital. Follow-up visits took place at day 7, 28 and 90. Blood samples were taken at day 0, 3, 7 and 28 and blood cultures for microbiology were taken at day 0 and day 7. Blood was cultured in BacTec Peds-Plus bottles (Becton Dickinson, NJ), incubated in the BD BACTEC™ FX40 (Becton Dickinson, Oxford, UK) for up to 7 days for detection of microbial growth. Positive cultures were Gram stained and sub-cultured to isolate different bacterial species. Gram positives were cultured on both differential (MacConkey agar plate) and enriched media (chocolate and blood agar plates) while Gram negatives were sub-cultured on chocolate agar. Bacterial identification and antibiotic susceptibility testing was done using the BD Phoenix M50 automated identification system (Becton Dickinson, Oxford, UK). Results were examined by a clinical officer and contaminants (*Micrococcus* species, *Staphylococcus epidermidis*/*saprophyticus*/*aureus* and *Bacillus* species) were treated as non-pathogens. Additional samples for PK were taken at 7 timepoints: approximately 1, 3 and 8 h after drug intake on day 0, before drug intake on day 2, 4 h and 18 h after intake on day 2, and at the day 7 follow-up visit.

### Data management and statistical analysis

Data was collected in case report forms (CRFs) and entered into an OpenClinica database. SAEs were reported on a standardised form and sent to the Clinical Trials Facility in Kilifi for review by a clinical officer. To assure the quality of the data, checks were performed in the database and by statisticians, and regular monitoring of the trial site and data was carried out by external monitors in Uganda.

Statistical analyses for the azithromycin intervention followed intention-to-treat, in order to better estimate efficacy under real-world conditions and reduce the risk of bias. The co-primary outcome of change in CRP at 72 h was analysed using linear regression adjusting for baseline values, based on complete cases. The composite measure of microbiological cure and 7-day survival was analysed as a binary outcome using logistic regression. Mortality secondary outcomes were analysed using Cox proportional hazards models and Kaplan–Meier curves. Length of stay was analysed as a time to event outcome (time from enrolment to discharge) treating death before discharge as a competing risk. Time from discharge to readmission was analysed using a competing risk model treating death before readmission as a competing risk. Fisher’s exact test was used to compare proportions of children having adverse events in each arm.

Children in the combined case and control groups were compared using Wilcoxon rank-sum tests for continuous variables and Fisher’s exact test for categorical variables.

### Pharmacokinetic-pharmacodynamic analysis

Azithromycin plasma concentrations were quantified using a validated liquid chromatography coupled with tandem mass spectrometry assay. The pharmacokinetic data were analysed using non-linear mixed effects compartmental pharmacokinetic models to describe the relationship between plasma concentrations as a function of time, dose and patient characteristics, assuming linear pharmacokinetics and log-normal distribution of inter- and intra-individual variability. The developed pharmacokinetic model was used to obtain the empirical Bayes estimates for the observed area under the curve during 24 h on day 3 (AUC_0-24h_) and the maximum concentration and time of maximum concentration of azithromycin in our population on day 3. An exploratory pharmacokinetic-pharmacodynamic analysis was performed by correlating CRP change with the empirical Bayes estimate for individual average azithromycin concentration from day 0 to day 3 as well as the maximum azithromycin concentration during this treatment period using Spearman’s rank-order.

### Pharmacokinetic analysis methods

Population pharmacokinetic (PK) analysis was performed by linear mixed-effect modelling using NONMEM version 7.5.1 (Icon plc, Dublin, Ireland) and Pirana 2.9.9 (Certara, Princeton, NJ), incorporating the ADVAN9 subroutine and first order conditional estimation with interaction (FOCE), with interaction when required. Additional statistical analysis and graphical quality review was performed with R Statistics 3.6.3 [20], using goodness-of-fit plots and prediction-corrected visual predictive checks (pcVPC) [21]. To account for the known effect of body size on pharmacokinetic parameters, the pharmacokinetic parameters were allometrically scaled a priori to a total body weight of 70 kg. Flow and volume parameters were scaled with allometric coefficients of 0.75 and 1, respectively [22]. Inter-individual and inter-occasion variability was considered to be log-normally distributed. Additive, proportional and combined additive and proportional error models were evaluated to describe the residual error.

Route of administration (oral intake or through a nasogastric tube) and intestinal fatty acid binding protein (I-FABP) concentration were tested as binary covariates for biological availability. Increased circulating I-FABP concentrations were previously linked to enterocyte damage, with an I-FABP concentration higher than 5.6 ng/mL being associated with an increased mortality risk [23]. In this study, we used an I-FABP level of 5.6 ng/mL as a threshold above which severe malabsorption, resulting in decreased bioavailability, was expected to occur. Age was investigated as a continuous covariate for bioavailability and clearance. A drop in objective function value of 3.84 or higher, corresponding with a *p* value < 0.05, was considered statistically significant.

The final model was used in a series of simulations to predict the AUC_0-24h_ per dosing interval on day 3. Hereto, we used a virtual population of sub-Saharan children from which 850 individuals were sampled per WHO weight band [24]. In adults on an effective dose of 500 mg yields a mean AUC_0-24h_ of 3.45 mg*h/L on day 3, that is known to be effective [25]. Hence, AUC_0-24h_ was chosen as the population target for the dosing regimen to be developed for the paediatric population.

As the AUC is a known predictor for efficacy of azithromycin [26], Monte Carlo simulations were then performed to establish a rational dosing regimen for azithromycin in this population across harmonised weight bands proposed by the WHO [27], using an average AUC_0-24h_ of 3.45 mg*h/L on the third day of treatment as reference exposure, since this is associated with an effective dose in adults [25]. The individual doses were rounded to the nearest 50 mg to facilitate dosing in practice, based on tablet sizes of 100 mg. The pharmacokinetic results are described in more detail in the supplementary material.

### Results

**Fig. 1:**
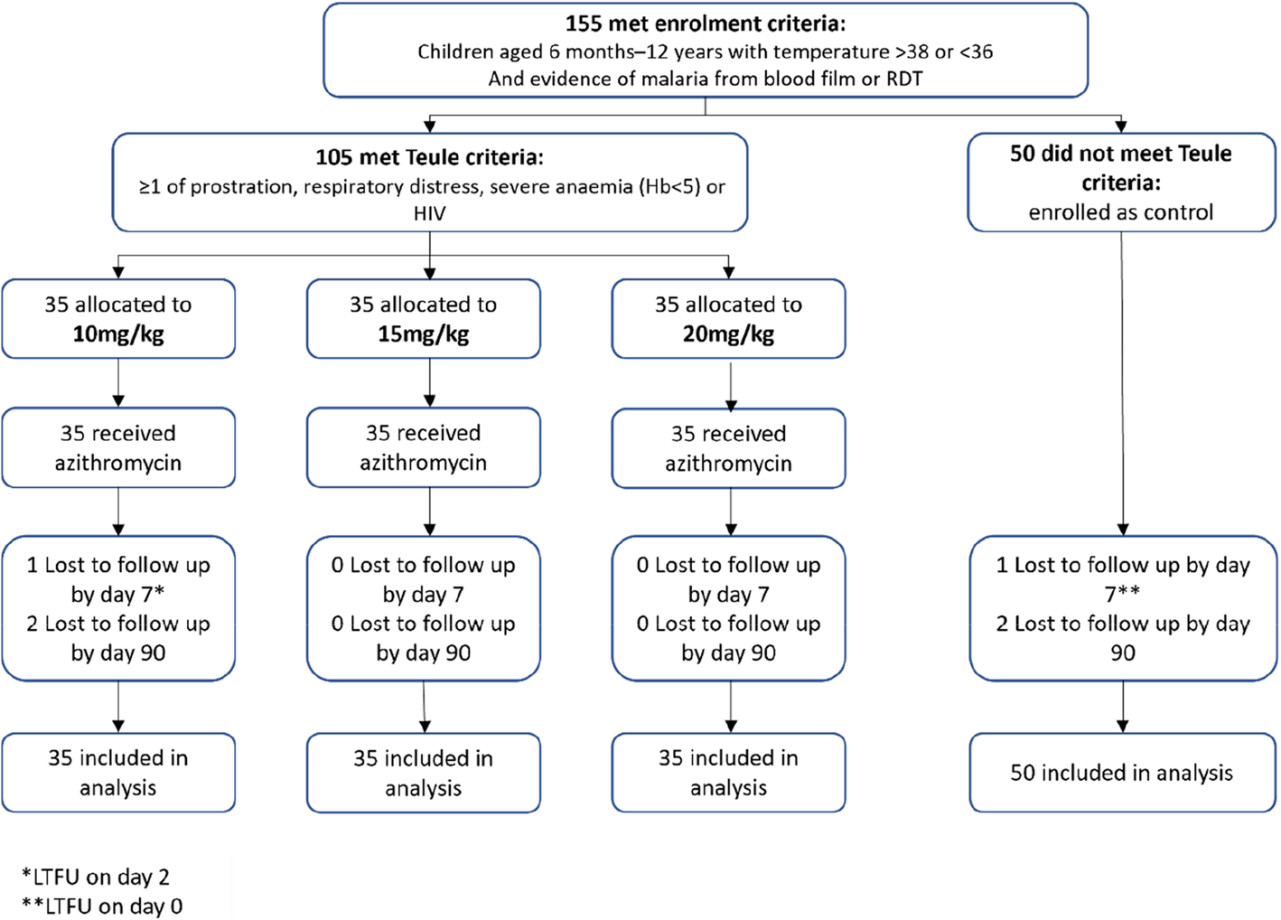
CONSORT diagram: trial flow

Enrolment for all participants began in January 2021. One hundred five cases who met the severity criteria were randomised to a dose of azithromycin: 35 to 10 mg/kg, 35 to 15 mg/kg and 35 to 20 mg/kg, with recruitment completed in October 2021 (Fig. 1). Enrolment of 50 controls without evidence of severe malaria was completed by June 2021. The final follow-up visit took place in January 2022.

### Baseline characteristics of cases

Baseline characteristics were fairly similar between the randomised arms although due to the small sample size some differences were present (Table 1; additional characteristics in Supplementary Table 2). The median age of cases was 66 months (IQR 38, 94). Most participants had a high temperature (81%), with a median of 38.3 °C (IQR 38.1, 38.7) while 19% had a temperature below 36 °C. Impaired consciousness was the most common of the severity criteria, being present in 74% of participants. The median score on the Blantyre Coma Scale among cases was 3 (IQR 3, 5) with only 5 children having a score of 2 or lower. Fifteen percent had a weak pulse, and almost a quarter (23%) reported having seizures in this illness. Almost half (47%) of cases had severe anaemia, with very few having increased work of breathing (defined as in-drawing or deep breathing) (4%) or HIV (1%). One hundred three out of 104 (99%) tested positive for malaria on the RDT, while 75/104 (72%) had a positive blood film. Of those that had a known malaria species, almost all had *P. falciparum* malaria, with another species found in only two children. The median *Pf*HRP2 was 278 pg/mL (IQR 0, 1342), with 27/101 (27%) having *Pf*HRP2 of 0, and the median CRP was 94 mg/L (IQR 42, 134). Only one case had a pathogen isolated from blood culture at baseline (*Enterococcus faecium*).

**Table 1:** Baseline characteristics

Characteristic/randomised arms	10 mg/kg (*N* = 35)	15 mg/kg (*N* = 35)	20 mg/kg (*N* = 35)	Cases (*N* = 105)	Control group (*N* = 50)
Sex—male	21 (60%)	16 (46%)	17 (49%)	54 (51%)	26 (52%)
Eligibility criteria					
Age at admission (months)	61 (34, 99)	66 (39, 94)	71 (38, 91)	66 (38, 94)	57 (29, 102)
Axillary temperature °C	38.3 (38.1, 38.7)	38.5 (38.2, 39.1)	38.3 (35.9, 38.6)	38.3 (38.1, 38.7)	38.2 (38.1, 38.8)
< 36 °C, *n* (%)	7 (20%)	3 (9%)	10 (29%)	20 (19%)	11 (22%)
Malaria tests^a^					
RDT—positive	34 (100%)	34 (97%)	35 (100%)	103 (99%)	49 (100%
RDT—negative	0 (0%)	1 (3%)	0 (0%)	1 (1%)	0 (0%)
Blood film—positive	22 (65%)	26 (74%)	27 (77%)	75 (72%)	40 (83%)
Blood film—negative	12 (35%)	9 (26%)	8 (23%)	29 (28%)	8 (17%)
Severity sign					
Impaired consciousness	25 (71%)	26 (74%)	27 (77%)	78 (74%)	0 (0%)
Increased work of breathing^b^	2 (6%)	2 (6%)	0 (0%)	4 (4%)	0 (0%)
Severe anaemia (Hb < 5 g/dL)	20 (57%)	13 (37%)	16 (46%)	49 (47%)	0 (0%)
Known HIV	0 (0%)	1 (3%)	0 (0%)	1 (1%)	0 (0%)
Other clinical results and malaria characteristics					
Haemoglobin (g/dL)	4.8 (3.8, 8.5)	6.0 (4.4, 9.7)	6.2 (4.2, 9.0)	5.8 (4.2, 9.0)	10.0 (8.7, 11.3)
Lactate (mmol/L)	2.3 (1.5, 3.6)	2.3 (1.5, 3.1)	2.3 (1.8, 2.9)	2.3 (1.6, 3.1)	2.0 (1.3, 2.9)
Blood glucose (mmol/L)	5.4 (4.6, 6.4)	5.5 (4.7, 6.4)	5.2 (4.3, 6.4)	5.3 (4.6, 6.4)	5.6 (4.7, 6.2)
Treatment in this illness					
Received oral antibiotics in the last week	1 (3%)	8 (26%)	7 (21%)	16 (16%)	11 (22%)
Malaria species	*N* = 22	*N* = 26	*N* = 27	*N* = 75	*N* = 40
* P. falciparum*	21 (95%)	26 (100%)	26 (96%)	73 (97%)	37 (93%)
Other	1 (5%)	0 (0%)	1 (4%)	2 (3%)	3 (8%)
HRP2	*N* = 34	*N* = 33	*N* = 34	*N* = 101	*N* = 48
HRP2 (pg/mL)	164 (0, 440)	329 (0, 2142)	511 (15, 2319)	278 (0, 1342)	199 (17, 654)
HRP2 = 0	10 (29%)	10 (30%)	7 (21%)	27 (27%)	7 (15%)
HRP2 > 1000 pg/mL	4 (12%)	11 (33%)	13 (38%)	28 (28%)	11 (23%)
CRP and microbiology					
CRP (mg/L)	82 (45, 129)	103 (46, 135)	90 (42, 133)	94 (42, 134)	66 (29, 89)
CRP (mg/L) mean (SD); *N*	84.3 (53.5); 32	93.6 (53.8); 34	93.2 (61.4); 35	90.5 (56.1); 101	68.5 (46.1); 49
Pathogen isolated	0 (0%)	1 (3%)	0 (0%)	1 (1%)	3 (6%)
Haemolytic streptococcus	0	0	0	0	1
* Enterococcus faecium*	0	1	0	1	0
Unidentified Gram-negative rod	0	0	0	0	2

Median (IQR) or *n* (%) unless otherwise stated

^a^One missing RDT and one missing blood film in 10 mg/kg arm, one RDT and two blood film not done in control arm. All children positive on either RDT or blood film

^b^Defined by in-drawing or deep breathing identified by staff

### Baseline characteristics of controls

Controls had a median age of 57 months (IQR 29, 102) and an axillary temperature of 38.2 °C (IQR 38.1, 38.8). Forty-nine out of 49 (100%) had a positive malaria RDT and 40/48 (83%) had a positive blood film. The median PfHRP2 was 199 pg/mL (IQR 17, 654) (*p* = 0.83 vs cases, Supplementary Table 3a) with 7/48 (15%) having PfHRP2 of 0. Controls had a lower CRP than cases, median 66 mg/L (29, 89) (*p* = 0.02). Pathogens were isolated in three controls (6%), with two reported as unidentified Gram-negative rods and the other haemolytic streptococcus.

### Adherence to protocol

Ninety-eight percent of children randomised to azithromycin received the correct dose according to the weight band dosing (Supplementary Table 1); two children received 50 mg more than expected due to being close to the top of the weight band. However, the doses received were still similar to the nominal dose (11 mg/kg for one child in the 10 mg/kg arm and 21.7 mg/kg for one child in the 20 mg/kg arm). No child reported stopping azithromycin early; 72 children were discharged before the fifth dose and continued to take azithromycin at home. Nine (9%) children needed at least one re-dose due to vomiting.

### Co-primary outcomes

**Fig. 2:**
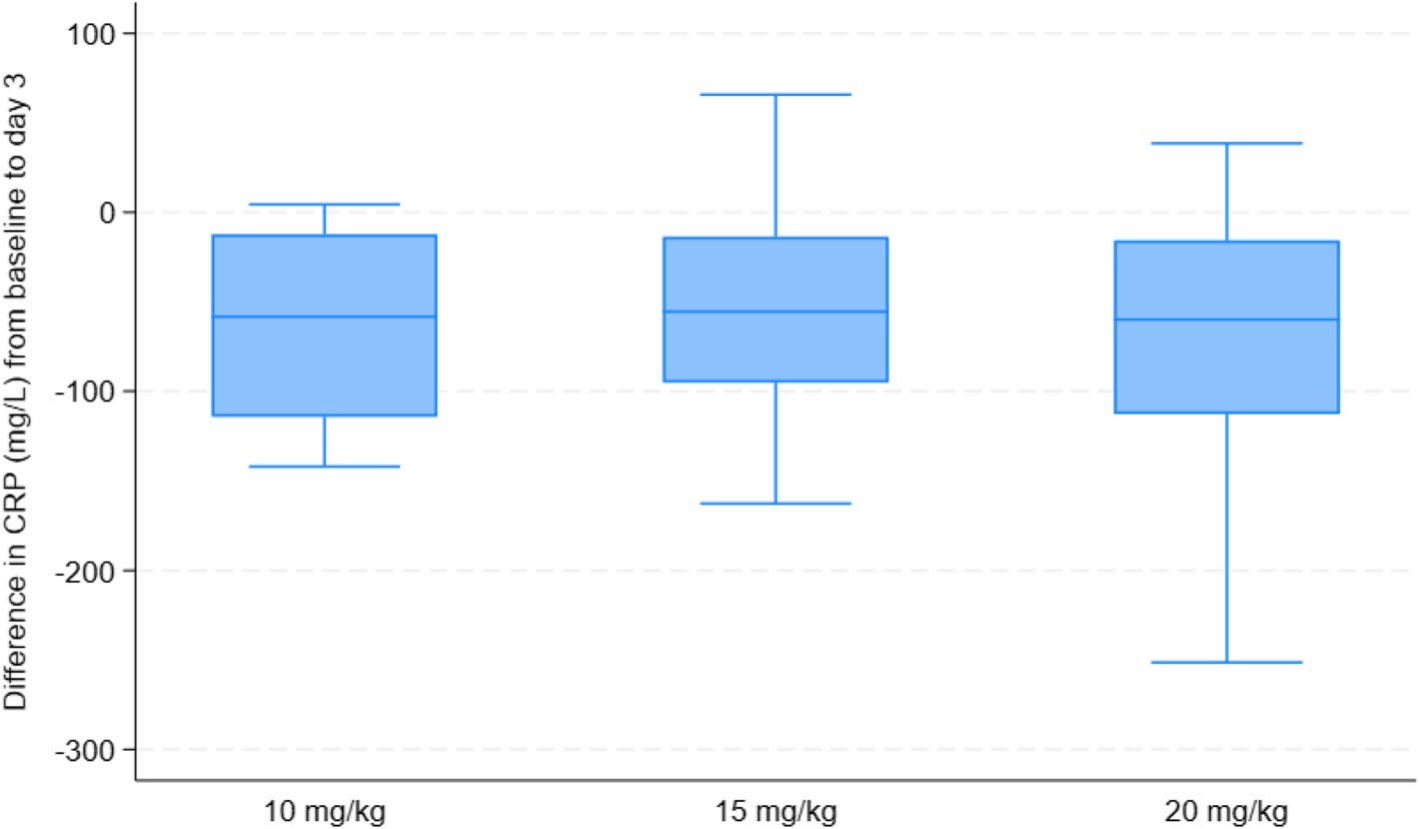
Boxplot of reductions in CRP in randomised trial arms

CRP reduced by 72 h in all arms (Table 2, Fig. 2). The mean change in the 10 mg/kg arm was − 65.8 mg/L (95% CI − 74.5, − 57.1); the change in 20 mg/kg was similar (− 64.8 mg/L (95% CI − 73.1, − 56.5); *p* = 0.87) while 15 mg/kg had a smaller reduction (− 51.2 (95% CI − 59.5, − 42.9); *p* = 0.02). Five children had missing CRP values at either day 0 or day 3.

**Table 2:** Primary and secondary outcomes

	**10 mg/kg**	**15 mg/kg**	**20 mg/kg**	**15 vs 10 estimate; *p* value**	**20 vs 10 estimate; *p* value**
Primary outcomes					
Change in CRP at 72 h—mean (95% CI)^a^	− 65.8 (− 74.5, − 57.1)	− 51.2 (− 59.5, − 42.9)	− 64.8 (− 73.1, − 56.5)	14.6 (2.6, 26.6); 0.02^b^	1.0 (− 11.0, 13.0); 0.87^b^
Deaths by 7 days—*n* (%)	1 (3%)	0 (0%)	1 (3%)		
Pathogen found at day 7—*n* (%)^c^	0 (0%)	0 (0%)	1^i^ (3%)		
Microbiological cure^d^					
7 day survival and microbiological cure				**–**	0.50 (0.04, 5.79); 0.58^e^
Secondary outcomes					
Mortality—*n* (%) cumulative deaths					
48 h	0 (0%)	0 (0%)	1 (3%)		
7 days	1 (3%)	0 (0%)	1 (3%)		1.00 (0.06, 15.99); 1.00^f^
28 days	1 (3%)	0 (0%)	1 (3%)		1.00 (0.06, 15.99); 1.00^f^
90 days	1 (3%)	1 (3%)	1 (3%)	0.96 (0.06, 15.37); 0.98^f^	0.98 (0.06, 15.67); 0.99^f^
Time to discharge—median (IQR) days	3 (3, 4)	3 (3, 4)	3 (3, 3)	1.08 (0.81, 1.43); 0.60^ g^	1.08 (0.78, 1.49); 0.66^ g^
Time to readmission—*n* (%) of children readmitted	2 (6%)	2 (6%)	3 (9%)	0.94 (0.13, 6.65); 0.95^ h^	1.40 (0.23, 8.60); 0.72^ h^

^a^Mean change in CRP between day 0 and day 3, estimated by linear regression adjusted for baseline values

^b^Mean difference between arms estimated by linear regression adjusted for baseline values

^c^Two children had a missing microbiology result at day 7

^d^The endpoint for microbiological cure was not calculated since only one pathogen was identified at baseline among cases

^e^Odds ratio estimated by logistic regression

^f^Hazard ratio from Cox proportional hazards model

^g^Subhazard ratio of time from randomisation to discharge from competing risks model with death before discharge treated as a competing risk

^h^Subhazard ratio of time from discharge to readmission from competing risks model with death before readmission treated as competing risk

^i^Haemolytic streptococcus

Due to only one pathogen being identified at baseline among cases, the planned outcome of microbiological cure alone was not analysed. For the composite outcome of 7-day survival and microbiological cure, 3 events in total contributed to the endpoint (2 deaths and 1 pathogen present at day 7), with no events in the 15 mg/kg arm. The odds ratio comparing 20 vs 10 mg/kg was 0.50 (95% CI 0.04, 5.79); *p* = 0.58.

### Secondary outcomes

One child in the 20 mg/kg arm died by 48 h (Table 2); as there were no other deaths by this timepoint, hazard ratios were not calculated. There was one additional death in the 10 mg/kg arm by day 7, with no evidence of a difference between 20 mg/kg vs 10 mg/kg to this timepoint (*p* = 1.00). No further deaths occurred by day 28. By day 90, there was one in each arm, resulting in similar 90-day survival across the arms (*p* = 0.98 and *p* = 0.99 for 15 and 20 mg/kg respectively vs 10 mg/kg).

All arms had a median length of stay of 3 days and similar time to discharge (Supplementary Fig. 2; vs 10 mg/kg, *p* = 0.60 for 15 mg/kg; *p* = 0.66 for 20 mg/kg). Seven cases were re-admitted by day 90 (Supplementary Fig. 3; 2 each in the 10 mg/kg and 15 mg/kg arms and 3 in the 20 mg/kg arm) with no evidence of differences in time to readmission (vs 10 mg/kg, *p* = 0.95 for 15 mg/kg; *p* = 0.72 for 20 mg/kg). No children were reported to have multiple readmissions.

There were 10 SAEs in total (Table 3); all were deaths or readmissions and no evidence of a difference between groups (vs 10 mg/kg, *p* = 1.00 for 15 mg/kg; *p* = 0.69 for 20 mg/kg). There were no additional grade 3/4 AEs reported other than the readmissions, and none of the AEs was related to azithromycin or led to a change in azithromycin.

**Table 3:** Adverse events

	**10 mg/kg**	**15 mg/kg**	**15 vs 10 *p* value**	**20 mg/kg**	**20 vs 10 *p* value**	**Control group**
SAEs						
Number of children ever having an SAE	3 (9%)	3 (9%)	1.00	4 (11%)	1.00	2 (4%)
Number of SAEs	3	3		4		2
SAE criteria						
Fatal	1 (33%)	1 (33%)		1 (25%)		0 (0%)
Life-threatening	0 (0%)	0 (0%)		0 (0%)		0 (0%)
Caused or prolonged hospitalisation	2 (67%)	2 (67%)		3 (75%)		2 (100%)
Persistent or significant disability/incapacity	0 (0%)	0 (0%)		0 (0%)		0 (0%)
Other important medical condition	0 (0%)	0 (0%)		0 (0%)		0 (0%)

No further grade 3/4 AEs were reported that were not SAEs. No SAEs were judged to be related to azithromycin, the dose of azithromycin, or lead to a change in azithromycin

### Pharmacokinetic-pharmacodynamic analysis

**Fig. 3:**
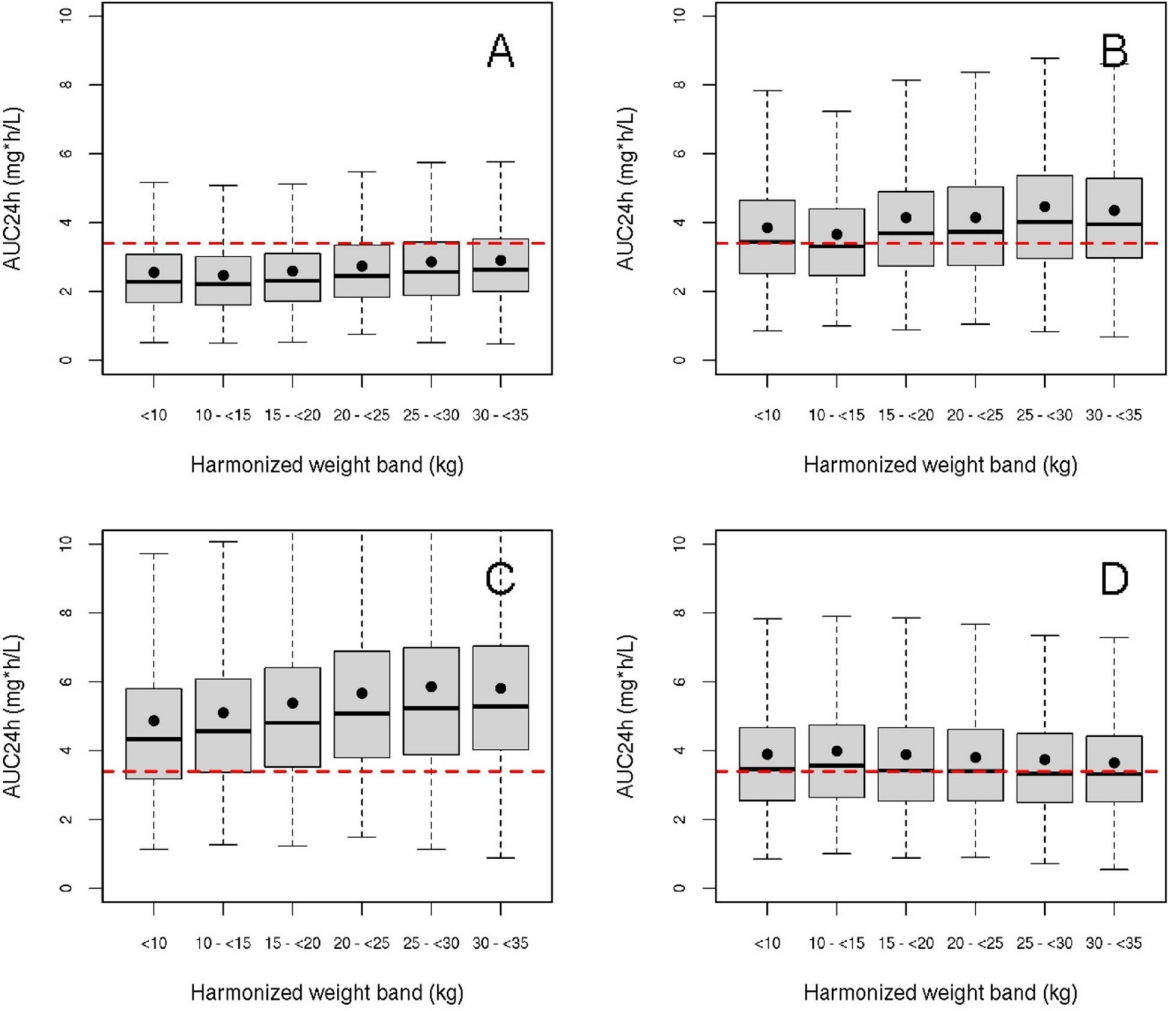
Comparison of pharmacokinetic parameters from conventional and allometric dosing regimens. Achieved exposure, reported as AUC_0-24 h_ on the third day of treatment, using different dosing regimens. The red dashed line indicates the mean exposure level of 3.45 mg*h/L in adults, that is used as a reference (25). The solid dots indicate the mean AUC_0-24 h_ resulting from the simulation. The boxes represent the predicted interquartile range and the solid black line represents the predicted median. The whiskers depict the extremes. **A** 10 mg/kg body weight; **B** 15 mg/kg body weight; **C** 20 mg/kg body weight; **D** allometric dosing, consisting of a flat dose per weight band according to Table 5. This dosing scheme results in a consistent exposure across all weight bands

A pharmacokinetic model with one oral absorption compartment and two disposition compartments, with a chain of 4 transition compartments to account for delayed onset of oral absorption, best described the data (details in Supplementary Fig. 4). Inter-occasion variability (IOV) in bioavailability was 71% (95% confidence interval: 60–85%). The empirical Bayes estimates for average concentration and maximum concentration since the last dose during the 3-day treatment period increased approximately linearly across the 10, 15 and 20 mg/kg arms (Table 4), with time to maximum concentration around 2.5–2.75 h. The maximum and average concentrations during this 3-day period did not correlate with absolute or relative reduction in C-reactive protein during treatment (Supplementary Fig. 7). An allometric dosing regimen, based on WHO weight bands (Table 5) resulted in consistent exposure across all weight bands in line with the 3.45 mg*h/L AUC_0-24h_ pharmacokinetic target for adults on day 3, in contrast to the 10, 15 and 20 mg/kg dosing regimens. The mg/kg dosing regimens tested in the trial resulted in relatively low predicted AUC_0-24h_ on day 3 in children with a low body weight and relative high exposure in children with a high body weight (Fig. 3A–C), whereas our alternative flat dose per WHO weight band, based on whole and half tablets equalised exposure without apparent increase in pharmacokinetic variability (Fig. 3D). The reference exposure on day 3 has been chosen as a surrogate for adequate exposure throughout the complete treatment period. Since azithromycin pharmacokinetics are linear, a dosing regimen that results in adequate exposure on day 3 will result in similar day 1 exposure as the reference exposure. The results for the predicted dosing regimens on different days than day 3 are given in Supplementary Fig. 8.

**Table 4:** Estimated PK characteristics of azithromycin after 3 days of treatment

Dose group (mg/kg)	C_max_ (mg/L)	CV (%)	T_max_ (h)	CV (%)	AUC_0-24 h_ (mg*h/L)	CV (%)
10	0.21	86	2.75	40	2.24	40
15	0.40	74	2.50	46	3.56	37
20	0.48	65	2.74	36	4.74	39

Values reported as geometric mean ± geometric coefficient of variation (CV). C_max_, maximum concentration; T_max_, time after dose at which maximum concentration is reached; AUC_0-24 h_, estimated area under the concentration time curve over 24 h on the third day of treatment

**Table 5:** Proposed allometric dosing scheme

**Harmonised weight band (kg)**	**Dose (mg)**	
6– < 10	150	1.5 tablets
10– < 15	200	2.0 tablets
15– < 20	250	2.5 tablets
20– < 25	300	3.0 tablets
25– < 30	350	3.5 tablets
30– < 35	400	4.0 tablets

Proposed dosing scheme, based on harmonised weight bands of the WHO and using 100 mg tablets

### Comparison of cases and controls at presentation to hospital

Due to the low number of pathogens found at admission, the planned analysis of factors which could identify those with bacterial infection in the combined cases and control group was not completed. Instead we analysed differences between the cases and controls in order to explore other factors which may be associated with bacterial co-infection. Factors found to differ significantly between cases and controls are presented in Supplementary Table 3a, and those with no evidence of difference in Supplementary Table 3b. Cases had significantly lower values for systolic blood pressure (median 90 vs 97; *p* < 0.001) and diastolic blood pressure (median 55 vs 59; *p* = 0.01). Cases were more likely to have received a blood transfusion both in this illness (30% vs 6% for controls; *p* < 0.001) and previously (44% vs 20%; *p* = 0.005) and were more likely to live in a rural area (90% vs 50%; *p* < 0.001). A higher proportion of cases had enlarged or gross splenomegaly (67% vs 34%; *p* < 0.001) and jaundice (34% vs 16%; *p* = 0.02). There were significant differences in many of the blood counts, with cases having higher white blood cells (median 9 vs 8 × 10^9^/L; *p* = 0.004), mean corpuscular volume (84 vs 81 fL; *p* = 0.002) and lymphocytes (3 vs 2 × 10^9^/L; *p* = 0.01) and lower red blood cells (2 vs 4 × 10^12^/L; *p* < 0.001). Cases had higher AST (52 vs 33 U/L; *p* < 0.001), bilirubin (1.1 vs 0.3 mg/dL; *p* < 0.001) and intestinal fatty acid binding protein (I-FABP) (1984 vs 1069 pg/mL; *p* = 0.01).

## Discussion

We have shown in a phase II clinical trial in Ugandan children with severe malaria that oral dispersible azithromycin, targeting bacterial co-infection, given alongside parenteral artesunate was safe and well tolerated (with only 9% of children requiring redosing for vomiting) for all three doses tested (10, 15 and 20 mg/kg). The mg/kg dosing worked well in practice with all children receiving their target doses within ± 2 mg/kg, but did not result in consistent exposure across all weight bands. The inclusion criteria we used to identify cases in this trial had previously identified a high risk group [7] for bacterial co-infection in Tanzania children with severe malaria; however, only one Gram-positive organism (*Enterococcus faecium*) was identified in the cases in our study, thus precluding any meaningful analysis with microbiological outcomes, alone or combined with survival, since mortality to day 7 was also low (2/105 (1.9%) children). Moreover, this also prevented us assessing our second objective to identify whether a combination of clinical, point-of-care diagnostic tests and/or biomarkers could accurately identify the sub-group of severe malaria with culture-proven bacteraemia. Notably, in our pharmacokinetic study we found that the estimated apparent oral clearance and volume parameters for azithromycin were roughly double to those previously reported by Zhao et al. [28] in children with uncomplicated malaria and Muto et al. [29] in children with respiratory tract infection. As both the apparent oral clearance and volume of a drug are inversely proportional to the fraction of drug absorbed, our findings suggest that the bioavailability of azithromycin in our population is reduced. The cause for this apparent difference in bioavailability needs further elucidation. Previously it has been found that antimalarial drug exposure may be increased during the acute phase of malaria [30], contrasting our results. Alternatively, critical illness may impair drug bioavailability [31] and severe illness in our population may, therefore, be a cause for our findings. Poor nutritional status or environmental enteropathy has been proposed to impact bioavailability of drugs by causing intestinal damage [32]. As described in our analysis, we exploratively investigated the correlation of I-FABP as a biomarker for intestinal damage with azithromycin [33]. These results have been described in the supplemental materials. This biomarker was found to be elevated in all study participants and we did not find an association between this biomarker and azithromycin pharmacokinetics. Our study was not designed to investigate potential causes for impaired bioavailability of azithromycin and we cannot rule out that intestinal damage due to either poor nutritional status or environmental enteropathy malnourishment impacted bioavailability.

One of the strengths of the study is the pharmacokinetic data on bioavailability of orally dispensed azithromycin in severe illness. We have shown that the studied dosing regimen, based according to a mg/kg dosing paradigm, does not lead to uniform exposure across all weight bands (Fig. 3), with relative under- and over-dosing in children with a low and high body weight, respectively. However, on average, the adult reference exposure on day 3 can be achieved using the 15 mg/kg dosing regimen (Table 5). This well-known phenomenon can be explained by the non-linear relationship between body size and clearance and can be resolved by accounting for this non-linear (allometric) relationship in the dosing regimen [34, 35]. We show that an allometric dosing regimen based on the harmonised WHO weight bands, rounded to the nearest 50 mg, reduces pharmacokinetic variability and is predicted to lead to effective exposure.

One of the limitations of our study was that it did not include many children under 24 months, thus the validity of the developed dosing regimen in this population could be debated. However, as the major elimination pathway of azithromycin is biliary excretion and maturation of this metabolic pathway occurs before 8 months [36], this suggests that the proposed allometric dosing regimen might reasonably be extrapolated to children as young as 8 months. In addition, owing to the low rate of bacteraemia in the cases we could not identify a relationship between systemic azithromycin exposure and CRP reduction in our study, as a proxy for microbiological cure (Supplementary Fig. 7)—one of the secondary objectives of the study. This lack of apparent pharmacokinetic-pharmacodynamic relationship could suggest no true association or indicate that the effect is already in the plateau of the dose–response curve with the doses tested. Given the heterogeneity of our population and pathogen susceptibility, as well as the relationship between CRP and both bacterial and malaria infection and simultaneous antimalarial and antibacterial treatment, we consider that a potential pharmacokinetic-pharmacodynamic relationship between both conditions may have been obscured. Henceforth, aiming for a target AUC_0-24 h_ of 3.45 mg*h/L on day 3 is currently the most appropriate dosing algorithm in absence of other pharmacokinetic-pharmacodynamic data.

Our study was also limited by a relatively small sample size, which had limited power to detect differences between study arms for our non-PK outcomes. The study was not powered to look at differences in clinical outcomes between study arm, but principally as a PKPD study to optimise dosing strategies for future trials, with the clinical data providing supporting information.

One potential reason that our study, using the same criteria in the Tanzanian study [7], failed to identify larger numbers with Gram-negative bacteraemia, specifically non-typhoidal salmonellae (NTS), could be that the median age of the Tanzanian study participants was 18 months (although the study did include infants) vs 66 months in TABS and the wider use of prereferral antibiotics (16%) in the cases. Risk factors for Gram-negative infection were explored in the Tanzanian study but it is unclear whether young age was not associated or not considered in the analysis. It is unknown whether the changing malaria epidemiology overtime, since the Tanzanian study was conducted, in which children of higher ages are presenting with severe malaria whose susceptibility to bacterial infection is unknown. However, another study conducted in Malawian children with a median age of 22 months (malaria status not reported) showed seasonality (specifically the wet/malaria season) coincided with peak incidence of NTS infection [37]. While our trial was conducted during a malaria season, it is likely that the temporal epidemiological changes in malaria prevalence in Eastern Uganda may have influenced underlying risk. This changing epidemiological risk over time has been elegantly demonstrated in a 9-year longitudinal case–control examining the relation between *P. falciparum* malaria and bacteraemia in Kenyan children involving 1454 cases with sickle cell trait HbAS, which confers strong protection against severe malaria, and 10,749 controls [5]. This found that at the beginning of the study, when malaria transmission was meso-hyperendemic (mean community parasite prevalence 29%), the bacteraemia incidence rate ratio associated with malaria parasitaemia was 6.69 (95% CI 1.31–34.3) in children aged 3 months–13 years, and 62% (8.2–91%) of bacteraemia cases occurred in children with malaria infection. HbAS was found to be strongly protective against hospital admission with bacteraemia. Over the 9-year period, the incidence of admission to hospital with malaria per 1000 child-years decreased from 28.5 to 3.45. The largest reduction was in the incidence of NTS bacteraemia, which was mirrored by a similar reduction in the protective effect of sickle cell trait against this organism.

With respect to severity of illness, we showed that a clear separation of risk factors in the cases compared to controls. With respect to *P. falciparu*m parasite burden measured by *Pf*HRP2, the Tanzanian children demonstrated those at greatest risk of bacteraemia were children with recent malaria, i.e. RDT positive but malaria slide negative and those with high parasite burdens. This observation is supported by a similar study in Kenya [38]. In TABS, 27% of children had no PfHRP2 (but a positive RDT) and 28/101 (28%) had PfHRP2 > 1000—so we would have expected to find more cases with concurrent bacteraemia. A systematic review found the severe malaria complication with the greatest risk of concurrent bacteraemia was severe anaemia [4]—a feature present in nearly half the cases in this trial.

The reason we opted for an oral formulation was that dispersible azithromycin was at the time of the study (originally funded in 2016) currently widely available at an affordable price, costing 0.40 euros per 100 mg oral dose. The dispersible formulation meant it could also be given by naso-gastric tube. Costs and cost-effectiveness are important considerations for any future intervention in children with severe malaria. Our study was not designed to study the effect of severe malabsorption on azithromycin pharmacokinetics. Nonetheless, we observed sufficient oral absorption of azithromycin in our very ill population, and we showed that therapeutic exposure can be attained using regular doses given orally. While the preference for parenteral antibiotics over oral dosing in severe illness has been challenged by lack of evidence [39], we cannot exclude the possibility, however, that in severe malaria there may be some reduced absorption. This may be caused by disordered gut barrier function, likely to be secondary to intense sequestration of parasitised red cells in the intestine [40–42] and/or acute severe perfusion injury secondary to severe anaemia or shock. To guide dosing of parenteral administration, the absolute bioavailability of azithromycin in our population should be investigated, as an apparent slightly decreased oral bioavailability was observed when compared to previous studies.

We had proposed that a future phase III trial could consider two strata [19]. The first in children at highest risk of bacterial co-infection would compare a pharmacologically informed dosage of oral azithromycin informed by the TABS trial to standard-of-care (largely third-generation cephalosporins). The second stratum would compare standard of care versus no antibiotics in children with severe malaria but minimal risk of bacterial co-infection. The trial’s objective would be to establish whether a policy for targeted antibiotic therapy could substantially reduce malaria-associated mortality while minimising the risks of excess antibiotic prescribing. Identifying the children at highest risk of co-infection for eligibility into the first strata we suggested could be determined by Teule criteria [7], but would also be informed by this pilot trial. However, we have not been able validate either the Teule criteria, other clinical criteria or biomarkers we proposed. The surrogate marker of putative bacterial co-infection, CRP, was elevated in both cases and controls and was significantly higher in cases. However, no differences between randomised arms were found in the day 7 outcomes, which largely normalised in all study arms (Supplementary Fig. 1). CRP may have been a more informative outcome measure if a higher rate of co-infection had been found. Intestinal fatty acid binding protein (I-FABP), a marker for acute enterocyte death, and putative intestinal permeability and endotoxin levels, has previously been demonstrated as elevated in severe malaria [43]; however, I-FABP testing is not currently available as a point of care diagnostic. Thus, it could be used in any future large study to stratify patients in the statistical analysis, but would be difficult to implement as eligibility criteria.

## Conclusions

We found no evidence of a difference in reduction of CRP from baseline, or microbiological cure or survival in children with severe malaria who took 10 mg/kg, 15 mg/kg or 20 mg/kg azithromycin orally over 5 days. In severe cases, we only identified one Gram-positive and no Gram-negative organisms at baseline, indicating that the inclusion criteria, based on a previous study in Teule, Tanzania, did not identify as many children with severe malaria at high risk of bacterial infection as we expected. Further work is needed in this area, potentially developing more point of care tests to better identify children at highest risk. We could not find evidence for an association between systemic azithromycin exposure and reduction in CRP, potentially due to the population enrolled to the trial. Weight band dosing may lead to more effective exposure.

## Supplementary Information

Supplementary Material 1.

## Data Availability

MRC CTU at UCL supports a controlled access approach based on completion of a data request proforma available from the lead author (r.connon@ucl.ac.uk).

## Abbreviations

AUC24h: Area under the curve at 24 h
AVPU: Alert, voice, pain, unresponsive: system of recording patient’s level of consciousness
CRF: Case report form
CRP: C-reactive protein
*E. coli*: *Escherichia coli*
ETAT: Emergency triage assessment and treatment
FEAST: Fluid Expansion As a Supportive Therapy
Hb: Haemoglobin
IBI: Invasive bacterial infection
ICREC: Imperial College Research Ethics Committee
KWTRP: KEMRI Wellcome Trust Research Programme
MRRH: Mbale Regional Referral Hospital
MRRH-REC: Mbale Regional Referral Hospital Research Ethics Committee
MRC: Medical Research Council
NTS: Non-typhoidal salmonella
PCT: Procalcitonin
PD: Pharmacodynamic
PfHRP2: *P. falciparum* HRP2 *Plasmodium falciparum* histidine-rich protein
PK: Pharmacokinetic
PKPD: Pharmacokinetic-pharmacodynamic
RDT: Rapid diagnostic test
TRACT: TRansfusion and Treatment strategies of severe Anaemia in African children Trial
WHO: World Health Organization
