# Supplementary material for: Azithromycin in severe malaria bacterial co-infection in African children (TABS-PKPD): a phase II randomised controlled trial
Source: BMC Med. 2024 Nov 6;22:516. doi: 10.1186/s12916-024-03712-5 (PMC11542398; doi:10.1186/s12916-024-03712-5)
Supplement: Supplementary file 1 — Supplementary Material 1. [file 12916_2024_3712_MOESM1_ESM.docx]

TABS Supplementary appendix

Contents

[Azithromycin Contraindications from the protocol 2](#_Toc179970690)

[Pharmacokinetic and pharmacodynamic analysis results 2](#_Toc179970691)

[Tables 3](#_Toc179970692)

[Supplementary Table 1: Weight band for mg/kg dosing dosing used in the TABS study 3](#_Toc179970693)

[Supplementary Table 2: Additional baseline characteristics 5](#_Toc179970694)

[Supplementary Table 3a: Baseline factors differing significantly between cases and controls 11](#_Toc179970695)

[Supplementary Table 3b: Additional comparison of cases and controls 12](#_Toc179970696)

[Supplementary Table 4: PK parameter estimates for the final model 17](#_Toc179970697)

[Figures 19](#_Toc179970698)

[Supplementary Figure 1: Follow up biochemistry and inflammatory markers 19](#_Toc179970699)

[Supplementary Figure 2: Days to discharge 22](#_Toc179970700)

[Supplementary Figure 3: Days from discharge to readmission 22](#_Toc179970701)

[Supplementary Figure 4: Schematic representation of the final PK model 23](#_Toc179970702)

[Supplementary Figure 5: Goodness of fit 24](#_Toc179970703)

[Supplementary Figure 6: Prediction-corrected visual predictive check 25](#_Toc179970704)

[Supplementary Figure 7: Reduction in CRP concentration versus azithromycin concentration 26](#_Toc179970705)

[Supplementary Figure 9: NONMEM control stream with final parameter estimates 28](#_Toc179970706)

[References 30](#_Toc179970707)

# Azithromycin Contraindications from the protocol

- Major contraindications to azithromycin, eg strong existing clinical diagnosis of QT-prolongation
- We referenced the UK BNF for children (https://bnfc.nice.org.uk/interactions/azithromycin/)

# Pharmacokinetic and pharmacodynamic analysis results

Guided by earlier studies, goodness-of-fit plots and a large drop in objective function value (OFV) relative to a one-compartment model, a two-compartment model was chosen as a base model with parameters clearance (CL), volume of the central compartment (V_2_), volume of the peripheral compartment (V_3_) and intercompartmental clearance (Q). The rapid absorption with a delay was described by implementing 4 absorption transit compartments[7]. The residual error was described with a proportional error model. There was no evidence that route of administration, IFABP concentrations and age improved model fit, and so these were not included in the final model (**Supplementary Table 4)**. A schematic representation of the pharmacokinetic model and parameter estimates are provided below (**Supplementary** **Figures S4-6**), together with, the final NONMEM control stream, including the parameter estimates **(Supplementary Figure 9)**.

# Tables

### Supplementary Table 1: Weight band for mg/kg dosing dosing used in the TABS study

|  | 10 mg/kg | 100mg tabs | 15 mg/kg | 100mg tabs | 20 mg/kg | 100mg tabs |
| --- | --- | --- | --- | --- | --- | --- |
| Weight (kg) | Weight x10 | Tab size | Weight x15 | Tab size | Weight x20 | Tab size |
| 5.5 - 6.4 | 55-64 | 0.5 | 82.5-97 | 1 | 110-129 | 1 |
| 6.5 - 7.4 | 65-74 | 0.5 | 97.5-112 | 1 | 130-149 | 1.5 |
| 7.5 - 8.4 | 75-84 | 0.5 | 112.5-127 | 1 | 150-169 | 1.5 |
| 8.5 - 9.4 | 85-94 | 1 | 127.5-142 | 1.5 | 170-189 | 1.5 |
| 9.5 - 10.4 | 95-104 | 1 | 142.5-157 | 1.5 | 190-209 | 2 |
| 10.5 - 11.4 | 105-114 | 1 | 157.5-172 | 1.5 | 210-229 | 2 |
| 11.5 - 12.4 | 115-124 | 1 | 172.5-187 | 1.5 | 230-249 | 2.5 |
| 12.5 - 13.4 | 125-134 | 1 | 187.5-202 | 2 | 250-269 | 2.5 |
| 13.5 - 14.4 | 135-144 | 1.5 | 202.5-217 | 2 | 270-289 | 2.5 |
| 14.5 - 15.4 | 145-154 | 1.5 | 217.5-232 | 2 | 290-309 | 3 |
| 15.5 -16.4 | 155-164 | 1.5 | 232.5-247 | 2.5 | 310-329 | 3 |
| 16.5 - 17.4 | 165-174 | 1.5 | 247.5-262 | 2.5 | 330-349 | 3.5 |
| 17.5 - 18.4 | 175-184 | 1.5 | 262.5-277 | 2.5 | 350-369 | 3.5 |
| 18.5 - 19.4 | 185-194 | 2 | 277.5-292 | 3 | 370-389 | 3.5 |
| 19.5 - 20.4 | 195-204 | 2 | 292.5-307 | 3 | 390-409 | 4 |
| 20.5 - 21.4 | 205-214 | 2 | 307.5-322 | 3 | 410-429 | 4 |
| 21.5 - 22.4 | 215-224 | 2 | 322.5-337 | 3 | 430-449 | 4.5 |
| 22.5 - 23.4 | 225-234 | 2 | 337.5-352 | 3.5 | 450-469 | 4.5 |
| 23.5 - 24.4 | 235-244 | 2.5 | 352.5-367 | 3.5 | 470-489 | 4.5 |
| 24.5 - 25.4 | 245-254 | 2.5 | 367.5-382 | 3.5 | 490-509 | 5 |
| 25.5 - 26.4 | 255-264 | 2.5 | 382.5-397 | 4 | 510-529 | 5 |
| 26.5 - 27.4 | 265-274 | 2.5 | 397.5-412 | 4 | 530-549 | 5.5 |
| 27.5 - 28.4 | 275-284 | 2.5 | 412.5-427 | 4 | 550-569 | 5.5 |
| 28.5 - 29.4 | 285-294 | 3 | 427.5-442 | 4.5 | 570-589 | 5.5 |
| 29.5 - 30.4 | 295-304 | 3 | 442.5-457 | 4.5 | 590-609 | 6 |
| 30.5 - 31.4 | 305-314 | 3 | 457.5-472 | 4.5 | 610-629 | 6 |

### Supplementary Table 2: Additional baseline characteristics

|  | **10mg/kg** | **15mg/kg** | **20mg/kg** | **Randomised arms total** | **Control group** |
| --- | --- | --- | --- | --- | --- |
| **Physical examination** | | | | | |
| Weight-for-age z-score | -0.9 (-1.5, -0.3) | -1.0 (-1.9, -0.4) | -1.1 (-1.9, -0.6) | -1.0 (-1.8, -0.4) | -0.7 (-1.2, 0.0) |
| MUAC-for-age z-score | -1.1 (-1.8, -0.1) | -1.4 (-1.8, -0.9) | -1.2 (-1.7, -0.5) | -1.2 (-1.8, -0.5) | -0.7 (-1.2, -0.3) |
| Weak pulse volume | 8 (23%) | 4 (11%) | 4 (11%) | 16 (15%) | 0 (0%) |
| Heart rate (bpm) | 142 (129, 153) | 144 (132, 153) | 134 (120, 148) | 141 (128, 152) | 136 (115, 155) |
| Systolic blood pressure (mmHg) | 89 (87, 94) | 90 (87, 97) | 91 (88, 98) | 90 (88, 96) | 97 (91, 100) |
| Oxygen saturation (%) | 98 (97, 99) | 98 (96, 99) | 98 (96, 99) | 98 (96, 99) | 98 (96, 99) |
| Blantyre Coma Score (BCS) | 4 (3, 5) | 3 (3, 5) | 3 (3, 5) | 3 (3, 5) | 5 (5, 5) |
| Seizures in this illness | 7 (20%) | 9 (26%) | 8 (23%) | 24 (23%) | 8 (16%) |
|  | | | | | |
| **Clinical history of this illness** | | | | | |
| History of fever | 33 (94%) | 35 (100%) | 33 (94%) | 101 (96%) | 48 (96%) |
| Duration of fever | 3 (3, 4) | 3 (3, 5) | 3 (2, 5) | 3 (3, 5) | 3 (2, 4) |
| History of cough | 22 (63%) | 17 (49%) | 13 (37%) | 52 (50%) | 24 (48%) |
| Increased work of breathing | 1 (3%) | 2 (6%) | 1 (3%) | 4 (4%) | 0 (0%) |
| Vomiting | 20 (57%) | 24 (69%) | 24 (69%) | 68 (65%) | 38 (76%) |
| Diarrhoea | 2 (6%) | 1 (3%) | 4 (11%) | 7 (7%) | 6 (12%) |
| Bloody diarrhoea | 0 (0%) | 0 (0%) | 1 (25%) | 1 (14%) | 0 (0%) |
| Haemoglobinuria | 15 (43%) | 7 (20%) | 11 (31%) | 33 (31%) | 10 (20%) |
| Days since haemoglobinuria started | 2 (1, 3) | 1 (1, 2) | 2 (1, 3) | 2 (1, 3) | 2 (1, 2) |
| Seizures in this illness | 7 (20%) | 9 (26%) | 8 (23%) | 24 (23%) | 8 (16%) |
| Seizures lasting more than 30 minutes | 1 (13%) | 1 (11%) | 1 (11%) | 3 (12%) | 0 (0%) |
| Inability to sit upright unsupported | 26 (74%) | 28 (80%) | 30 (86%) | 84 (80%) | 1 (2%) |
|  | | | | | |
| **Treatment in this illness** | | | | | |
| Admitted for over 24 hours into another hospital | 1 (3%) | 1 (3%) | 0 (0%) | 2 (2%) | 1 (2%) |
| Received oral antimalarials in the last week: Yes  No  Don't know | 20 (57%)  15 (43%)  0 (0%) | 14 (40%)  21 (60%)  0 (0%) | 14 (40%)  20 (57%)  1 (3%) | 48 (46%)  56 (53%)  1 (1%) | 26 (52%)  24 (48%)  0 (0%) |
| Received a blood transfusion | 13 (37%) | 9 (26%) | 9 (26%) | 31 (30%) | 3 (6%) |
| Received oral antibiotics in the last week | 1 (3%) | 8 (26%) | 7 (21%) | 16 (16%) | 11 (22%) |
| Number of doses of IV or IM quinine/artesunate | 0 (0, 0) | 0 (0, 1) | 0 (0, 1) | 0 (0, 1) | 0 (0, 0) |
|  | | | | | |
| **Past history before this illness** | | | | | |
| Two or more hospital admissions in the last year: Yes  No  Don't know | 9 (26%)  26 (74%)  0 (0%) | 8 (23%)  27 (77%)  0 (0%) | 12 (34%)  22 (63%)  1 (3%) | 29 (28%)  75 (71%)  1 (1%) | 19 (38%)  31 (62%)  0 (0%) |
| Previously received a blood transfusion: Yes  No  Don't know | 15 (43%)  20 (57%)  0 (0%) | 17 (49%)  18 (51%)  0 (0%) | 14 (40%)  20 (57%)  1 (3%) | 46 (44%)  58 (55%)  1 (1%) | 10 (20%)  40 (80%)  0 (0%) |
| Number of occasions:  1  2  3-4  5+ | 3 (9%)  2 (6%)  2 (6%)  8 (23%) | 5 (14%)  3 (9%)  5 (14%)  4 (11%) | 5 (14%)  0 (0%)  2 (6%)  7 (20%) | 13 (12%)  5 (5%)  9 (9%)  19 (18%) | 4 (8%)  1 (2%)  2 (4%)  3 (6%) |
| Epilepsy | 0 (0%) | 0 (0%) | 0 (0%) | 0 (0%) | 0 (0%) |
| Able to sit without support | 32 (91%) | 33 (94%) | 34 (97%) | 99 (94%) | 48 (96%) |
| Able to walk without help | 30 (86%) | 32 (91%) | 32 (91%) | 94 (90%) | 45 (90%) |
|  | | | | | |
| **Family details** | | | | | |
| Number of siblings | 3 (2, 5) | 3 (2, 5) | 3 (1, 6) | 3 (2, 5) | 3 (1, 4) |
| Parents still alive:  Both alive  One alive  Both died | 35 (100%)  0 (0%)  0 (0%) | 33 (94%)  2 (6%)  0 (0%) | 35 (100%)  0 (0%)  0 (0%) | 103 (98%)  2 (2%)  0 (0%) | 49 (98%)  1 (2%)  0 (0%) |
| Homestead where child lives: Urban  Semi-urban  Rural | 1 (3%)  2 (6%)  32 (91%) | 1 (3%)  2 (6%)  32 (91%) | 2 (6%)  2 (6%)  31 (89%) | 4 (4%)  6 (6%)  95 (90%) | 3 (6%)  22 (44%)  25 (50%) |
| Sleeps under mosquito net | 33 (94%) | 33 (94%) | 32 (91%) | 98 (93%) | 50 (100%) |
|  | | | | | |
| **Clinical examination** | | | | | |
| Indrawing | 0 (0%) | 1 (3%) | 0 (0%) | 1 (1%) | 0 (0%) |
| Deep breathing | 0 (0%) | 0 (0%) | 0 (0%) | 0 (0%) | 0 (0%) |
| Crackles: Unilateral  Bilateral  None | 0 (0%)  0 (0%)  35 (100%) | 1 (3%)  0 (0%)  34 (97%) | 0 (0%)  0 (0%)  35 (100%) | 1 (1%)  0 (0%)  104 (99%) | 1 (2%)  1 (2%)  48 (96%) |
| Sunken eyes | 1 (3%) | 0 (0%) | 0 (0%) | 1 (1%) | 0 (0%) |
| Decreased skin turgor | 0 (0%) | 0 (0%) | 0 (0%) | 0 (0%) | 0 (0%) |
| Cold hands or feet | 0 (0%) | 0 (0%) | 0 (0%) | 0 (0%) | 0 (0%) |
| Liver size >2cm below costal margin | 5 (14%) | 10 (29%) | 3 (9%) | 18 (17%) | 3 (6%) |
| Splenomegaly:  Not palpable  Enlarged  Gross | 10 (29%)  24 (69%)  1 (3%) | 15 (43%)  20 (57%)  0 (0%) | 10 (29%)  24 (69%)  1 (3%) | 35 (33%)  68 (65%)  2 (2%) | 33 (66%)  14 (28%)  3 (6%) |
| Jaundice | 12 (34%) | 9 (26%) | 15 (43%) | 36 (34%) | 8 (16%) |
| Very severe wasting | 0 (0%) | 0 (0%) | 0 (0%) | 0 (0%) | 0 (0%) |
| Kwashiorkor: None  Pretibial  Hands/legs  Generalised | 35 (100%)  0 (0%)  0 (0%)  0 (0%) | 35 (100%)  0 (0%)  0 (0%)  0 (0%) | 35 (100%)  0 (0%)  0 (0%)  0 (0%) | 105 (100%)  0 (0%)  0 (0%)  0 (0%) | 50 (100%)  0 (0%)  0 (0%)  0 (0%) |
| Generalised lymphadenopathy | 0 (0%) | 0 (0%) | 0 (0%) | 0 (0%) | 0 (0%) |
| Flaky paint dermatitis | 0 (0%) | 0 (0%) | 0 (0%) | 0 (0%) | 0 (0%) |
| Oral candidiasis | 0 (0%) | 0 (0%) | 0 (0%) | 0 (0%) | 0 (0%) |
|  | | | | | |
| **Neurological** | | | | | |
| Fitting currently | 1 (3%) | 1 (3%) | 1 (3%) | 3 (3%) | 0 (0%) |
| Neck stiffness | 0 (0%) | 0 (0%) | 0 (0%) | 0 (0%) | 0 (0%) |
| Bulging fontanelle (infants only): Yes  No  Not assessed | 0 (0%)  23 (66%)  12 (34%) | 0 (0%)  17 (49%)  18 (51%) | 0 (0%)  19 (54%)  16 (46%) | 0 (0%)  59 (56%)  46 (44%) | 0 (0%)  39 (78%)  11 (22%) |
| Pupil symmetry: Unequal | 0 (0%) | 0 (0%) | 0 (0%) | 0 (0%) | 0 (0%) |
| Abnormal motor posturing | 0 (0%) | 0 (0%) | 1 (3%) | 1 (1%) | 0 (0%) |
|  | | | | | |
| **Presentation** | | | | | |
| Facility child first presented to:  Study hospital  Level II  Level III  Level IV  Other district hospital  Private hospital | 28 (80%)  0 (0%)  1 (3%)  3 (9%)  1 (3%)  2 (6%) | 30 (86%)  0 (0%)  0 (0%)  2 (6%)  1 (3%)  2 (6%) | 30 (86%)  0 (0%)  1 (3%)  1 (3%)  2 (6%)  1 (3%) | 88 (84%)  0 (0%)  2 (2%)  6 (6%)  4 (4%)  5 (5%) | 46 (92%)  2 (4%)  0 (0%)  2 (4%)  0 (0%)  0 (0%) |
| Time to enrolment since presented at other facility | 16 (7, 27) | 21 (16, 26) | 5 (2, 7) | 12 (6, 25) | ² |
| Time to enrolment since referred from other facility | 5 (4, 16) | 5 (4, 7) | 2 (1, 3) | 4 (3, 6) | ² |
|  | | | | | |
| **Admission blood tests** | | | | | |
| Malaria pigment –  Yes  No  Not done | 8 (24%)  25 (74%)  1 (3%) | 7 (20%)  28 (80%)  0 (0%) | 5 (14%)  30 (86%)  0 (0%) | 20 (19%)  83 (80%)  1 (1%) | 6 (12%)  42 (84%)  2 (4%) |
| Malaria parasite count per μL | 39580 (579, 112880) | 36790 (563, 149200) | 6134 (647, 108000) | 26160 (579, 127920) | 16140 (1048, 126540) |
| WBC (10⁹/L) | 11 (8, 18) | 9 (6, 21) | 9 (7, 16) | 9 (7, 18) | 8 (7, 9) |
| RBC (10¹²/L) | 2 (2, 3) | 3 (2, 4) | 2 (2, 4) | 2 (2, 4) | 4 (3, 4) |
| MCV (fL) | 84 (79, 96) | 86 (79, 94) | 84 (77, 88) | 84 (79, 92) | 81 (75, 86) |
| MCH (picograms) | 28 (26, 30) | 27 (26, 30) | 27 (25, 29) | 27 (25, 30) | 26 (24, 28) |
| MCHC (g/dL) | 32 (31, 34) | 31 (31, 34) | 32 (31, 34) | 32 (31, 34) | 33 (32, 34) |
| Platelets (10⁹/L) | 173 (124, 261) | 150 (82, 304) | 165 (114, 261) | 158 (99, 261) | 184 (132, 239) |
| Lymphocytes (10⁹/L) | 3 (2, 5) | 4 (2, 7) | 3 (2, 6) | 3 (2, 6) | 2 (2, 4) |
| Neutrophils (10⁹/L) | 6 (4, 8) | 5 (3, 9) | 5 (3, 9) | 5 (4, 9) | 4 (3, 5) |
| Monocytes (10⁹/L) | 1 (0, 1) | 1 (0, 1) | 1 (0, 2) | 1 (0, 2) | 1 (1, 1) |
| Sodium (mmol/L) | 135 (130, 140) | 134 (126, 143) | 139 (131, 152) | 136 (130, 144) | 131 (126, 137) |
| Potassium (mmol/L) | 5 (4, 5) | 4 (4, 5) | 4 (4, 5) | 4 (4, 5) | 4 (4, 5) |
| Urea/BUN (mmol/L) | 5 (3, 13) | 4 (3, 7) | 4 (3, 11) | 5 (3, 9) | 4 (3, 5) |
| Creatinine (mg/dL) | 0 (0, 1) | 0 (0, 1) | 0 (0, 1) | 0 (0, 1) | 0 (0, 1) |
| Albumin (g/L) | 38 (34, 41) | 40 (34, 43) | 38 (33, 41) | 38 (34, 41) | 40 (36, 42) |
| AST (U/L) | 58 (32, 117) | 49 (33, 75) | 46 (24, 98) | 52 (32, 103) | 33 (26, 44) |
| ALT (U/L) | 15 (9, 20) | 16 (10, 21) | 10 (8, 18) | 13 (9, 20) | 12 (7, 15) |
| Bilirubin (mg/dL) | 1 (0, 3) | 1 (0, 2) | 1 (0, 2) | 1 (0, 2) | 0 (0, 1) |
| PCT (pg/mL): N (%) greater than 0 | 6 (18%) | 6 (17%) | 6 (18%) | 18 (17%) | 4 (8%) |
| PCT (pg/mL): Median (IQR) all values | 0 (0, 0) | 0 (0, 0) | 0 (0, 0) | 0 (0, 0) | 0 (0, 0) |
| PCT (pg/mL): Median (IQR) excluding 0 | 710 (228, 3510) | 201 (84, 306) | 192 (99, 1096) | 245 (144, 1052) | 280 (120, 490) |
| IFABP (pg/mL) | 1997 (1390, 2792) | 1715 (1002, 4008) | 2377 (1109, 4736) | 1984 (1120, 3897) | 1069 (444, 2485) |

### Supplementary Table 3a: Baseline factors differing significantly between cases and controls

|  | Case (N=105) | Control (N=50) | p-value^1^ |
| --- | --- | --- | --- |
| **Clinical or demographic characteristics** |  |  |  |
| Impaired consciousness | 78 (74%) | 0 (0%) | <0.001 |
| Severe anaemia (Hb<5g/dL) | 49 (47%) | 0 (0%) | <0.001 |
| Systolic blood pressure (mmHg) | 90 (88, 96) | 97 (91, 100) | <0.001 |
| Diastolic blood pressure (mmHg) | 55 (51, 60) | 59 (53, 64) | 0.01 |
| Capillary refill time > 3 seconds | 1 (1, 2) | 1 (1, 1) | <0.001 |
| Weak radial pulse volume | 16 (15%) | 0 (0%) | 0.002 |
| Blantyre Coma Score (BCS) | 3 (3, 5) | 5 (5, 5) | <0.001 |
| Inability to sit upright unsupported | 84 (80%) | 1 (2%) | <0.001 |
| Received a blood transfusion in this illness | 31 (30%) | 3 (6%) | <0.001 |
| Previously received a blood transfusion | 46 (44%) | 10 (20%) | 0.005 |
| Homestead where child lives – rural | 95 (90%) | 25 (50%) | <0.001 |
| Splenomegaly – enlarged or gross | 70 (67%) | 17 (34%) | <0.001 |
| Jaundice | 36 (34%) | 8 (16%) | 0.02 |
| **Lab results** |  |  |  |
| Haemoglobin (g/dL) | 5.8 (4.2, 9.0) | 10.0 (8.7, 11.3) | <0.001 |
| White blood cells (10⁹/L) | 9 (7, 18) | 8 (7, 9) | 0.004 |
| Red blood cells (10¹²/L) | 2 (2, 4) | 4 (3, 4) | <0.001 |
| MCV (fL) | 84 (79, 92) | 81 (75, 86) | 0.002 |
| MCH (picograms) | 27 (25, 30) | 26 (24, 28) | 0.04 |
| MCHC (g/dL) | 32 (31, 34) | 33 (32, 34) | 0.04 |
| Lymphocytes (10⁹/L) | 3 (2, 6) | 2 (2, 4) | 0.01 |
| Neutrophils (10⁹/L) | 5 (4, 9) | 4 (3, 5) | 0.01 |
| Sodium (mmol/L) | 136 (130, 144) | 131 (126, 137) | 0.02 |
| AST (U/L) | 52 (32, 103) | 33 (26, 44) | <0.001 |
| Bilirubin (mg/dL) | 1.1 (0.3, 1.8) | 0.3 (0.1, 0.8) | <0.001 |
| CRP (mg/L) | 94 (42, 134) | 66 (29, 89) | 0.02 |
| IFABP (pg/mL) | 1984 (1120, 3897) | 1069 (444, 2485) | 0.01 |

^1^P-value from Wilcoxon rank-sum tests for continuous variables, and Fisher’s exact tests for categorical variables.

### Supplementary Table 3b: Additional comparison of cases and controls

|  | **Case** | **Control** | **p-value^1^** |
| --- | --- | --- | --- |
| Number enrolled | 105 | 50 |  |
| Sex – male | 54 (51%) | 26 (52%) | 1.00 |
|  | | | |
| **Inclusion criteria** | | | |
| Age at admission (months) | 66 (38, 94) | 57 (29, 102) | 0.67 |
| Axillary temperature °C | 38.3 (38.1, 38.7) | 38.2 (38.1, 38.8) | 0.25 |
| Increased work of breathing | 4 (4%) | 0 (0%) | 0.31 |
| Known HIV | 1 (1%) | 0 (0%) | 1.00 |
|  | | | |
| **Physical examination** | | | |
| Heart rate (bpm) | 141 (128, 152) | 136 (115, 155) | 0.28 |
| Respiration rate (breaths per minute) | 36 (31, 44) | 35 (30, 40) | 0.23 |
| Oxygen saturation (%) | 98 (96, 99) | 98 (96, 99) | 0.16 |
| Temperature gradient | 2 (2%) | 1 (2%) | 1.00 |
|  | | | |
| **Bedside tests at admission** | | | |
| Lactate (mmol/L) | 2.3 (1.6, 3.1) | 2.0 (1.3, 2.9) | 0.13 |
| Blood glucose (mmol/L) | 5.3 (4.6, 6.4) | 5.6 (4.7, 6.2) | 0.48 |
|  | | | |
| **Clinical history of this illness** | | | |
| History of fever | 101 (96%) | 48 (96%) | 1.00 |
| Duration of fever | 3 (3, 5) | 3 (2, 4) | 0.22 |
| History of cough | 52 (50%) | 24 (48%) | 0.87 |
| Increased work of breathing | 4 (4%) | 0 (0%) | 0.31 |
| Vomiting | 68 (65%) | 38 (76%) | 0.20 |
| Diarrhoea | 7 (7%) | 6 (12%) | 0.35 |
| Bloody diarrhoea | 1 (14%) | 0 (0%) | 1.00 |
| Haemoglobinuria | 33 (31%) | 10 (20%) | 0.18 |
| Days since haemoglobinuria started | 2 (1, 3) | 2 (1, 2) | 0.64 |
| Seizures in this illness | 24 (23%) | 8 (16%) | 0.40 |
| Seizures lasting more than 30 minutes | 3 (12%) | 0 (0%) | 1.00 |
|  | | | |
| **Treatment in this illness** | | | |
| Admitted for over 24 hours into another hospital | 2 (2%) | 1 (2%) | 1.00 |
| Received oral antimalarials in the last week: Yes  No  Don't know | 48 (46%)  56 (53%)  1 (1%) | 26 (52%)  24 (48%)  0 (0%) | 0.73 |
| Received oral antibiotics in the last week | 16 (16%) | 11 (22%) | 0.50 |
| Number of doses of IV or IM quinine/artesunate | 0 (0, 1) | 0 (0, 0) | 0.88 |
|  | | | |
| **Past history before this illness** | | | |
| Two or more hospital admissions in the last year: Yes  No  Don't know | 29 (28%)  75 (71%)  1 (1%) | 19 (38%)  31 (62%)  0 (0%) | 0.41 |
| Number of occasions: 1  2  3-4  5+ | 13 (12%)  5 (5%)  9 (9%)  19 (18%) | 4 (8%)  1 (2%)  2 (4%)  3 (6%) | 0.95 |
| Epilepsy | 0 (0%) | 0 (0%) |  |
| Able to sit without support | 99 (94%) | 48 (96%) | 1.00 |
| Able to walk without help | 94 (90%) | 45 (90%) | 1.00 |
|  | | | |
| **Family details** | | | |
| Number of siblings | 3 (2, 5) | 3 (1, 4) | 0.11 |
| Parents still alive:  Both alive  One alive  Both died | 103 (98%)  2 (2%)  0 (0%) | 49 (98%)  1 (2%)  0 (0%) | 1.00 |
| Sleeps under mosquito net | 98 (93%) | 50 (100%) | 0.10 |
|  | | | |
| **Clinical examination** | | | |
| Indrawing | 1 (1%) | 0 (0%) | 1.00 |
| Deep breathing | 0 (0%) | 0 (0%) | 1.00 |
| Crackles: Unilateral  Bilateral  None | 1 (1%)  0 (0%)  104 (99%) | 1 (2%)  1 (2%)  48 (96%) | 0.24 |
| Sunken eyes | 1 (1%) | 0 (0%) | 1.00 |
| Decreased skin turgor | 0 (0%) | 0 (0%) | 1.00 |
| Cold hands or feet | 0 (0%) | 0 (0%) | 1.00 |
| Liver size >2cm below costal margin | 18 (17%) | 3 (6%) | 0.08 |
| Very severe wasting | 0 (0%) | 0 (0%) | 1.00 |
| Kwashiorkor: None  Pretibial  Hands/legs  Generalised | 105 (100%)  0 (0%)  0 (0%)  0 (0%) | 50 (100%)  0 (0%)  0 (0%)  0 (0%) | 1.00 |
| Generalised lymphadenopathy | 0 (0%) | 0 (0%) | 1.00 |
| Flaky paint dermatitis | 0 (0%) | 0 (0%) | 1.00 |
| Oral candidiasis | 0 (0%) | 0 (0%) | 1.00 |
|  | | | |
| **Neurological** | | | |
| Fitting currently | 3 (3%) | 0 (0%) | 0.55 |
| Neck stiffness | 0 (0%) | 0 (0%) | 1.00 |
| Pupil symmetry: Unequal | 0 (0%) | 0 (0%) | 1.00 |
| Abnormal motor posturing | 1 (1%) | 0 (0%) | 1.00 |
|  | | | |
| **Presentation** | | | |
| Facility child first presented to:  Study hospital  Level II  Level III  Level IV  Other district hospital  Private hospital | 88 (84%)  0 (0%)  2 (2%)  6 (6%)  4 (4%)  5 (5%) | 46 (92%)  2 (4%)  0 (0%)  2 (4%)  0 (0%)  0 (0%) | 0.10 |
| Time to enrolment since presented at other facility | 12 (6, 25) | ² |  |
| Time to enrolment since referred from other facility | 4 (3, 6) | ² |  |
| **Admission blood tests and admission microbiology** | | | |
| Malaria RDT - Positive  Negative  Not done/invalid | 103 (98%)  1 (1%)  1 (1%) | 49 (98%)  0 (0%)  1 (2%) | 0.69 |
| Malaria blood film - Positive  Negative  Not done/invalid | 75 (71%)  29 (28%)  1 (1%) | 40 (80%)  8 (16%)  2 (4%) | 0.12 |
| Malaria pigment - Yes  No  Not done | 20 (19%)  83 (80%)  1 (1%) | 6 (12%)  42 (84%)  2 (4%) | 0.21 |
| Malaria species -  P. falciparum  P. malariae  P. ovale  P. vivax  Not done/missing | 73 (70%)  0 (0%)  1 (1%)  1 (1%)  30 (29%) | 37 (74%)  0 (0%)  3 (6%)  0 (0%)  10 (20%) | 0.17 |
| Malaria parasite count per μL | 26160 (579, 127920) | 16140 (1048, 126540) | 0.75 |
| HRP2 (pg/ml) | 278 (0, 1342) | 199 (17, 654) | 0.83 |
| Platelets (10⁹/L) | 158 (99, 261) | 184 (132, 239) | 0.64 |
| Monocytes (10⁹/L) | 1 (0, 2) | 1 (1, 1) | 0.53 |
| Potassium (mmol/L) | 4 (4, 5) | 4 (4, 5) | 0.17 |
| Creatinine (mg/dL) | 0 (0, 1) | 0 (0, 1) | 0.89 |
| Albumin (g/L) | 38 (34, 41) | 40 (36, 42) | 0.18 |
| ALT (U/L) | 13 (9, 20) | 12 (7, 15) | 0.09 |
| PCT (pg/mL): greater than 0 | 18 (17%) | 4 (8%) | 0.15 |
| PCT (pg/mL): Median (IQR) all values | 0 (0, 0) | 0 (0, 0) | 0.12 |
| PCT (pg/mL): Median (IQR) excluding 0 | 245 (144, 1052) | 280 (120, 490) | 0.73 |
| Pathogen isolated | 1 (1%) | 3 (6%) | 0.10 |
| Pathogen -  Haemolytic streptococcus  Enterococcus faecium  Unidentified Gram-negative rod | 0 (0%)  1 (100%)  0 (0%) | 1 (33%)  0 (0%)  2 (67%) | 0.50 |

^1^ P-value from Wilcoxon rank-sum tests for continuous variables, and Fisher’s exact tests for categorical variables.

### Supplementary Table 4: PK parameter estimates for the final model

| **Parameter (unit)** | **Value** | **95% CI** | | |
| --- | --- | --- | --- | --- |
| CL/F (L/h) | 181 | 146 - 206 | | |
| Vc/F (L) | 3140 | 2416 - 4085 | | |
| MAT (h) | 2.35 | 2.06 - 2.67 | | |
| Q/F (L/h) | 422 | 346 - 536 | | |
| Vp/F (L) | 6280 | 4209 - 9592 | | |
|  |  |  |  |  |
| IIV on Vc (%CV) | 98 | 67 – 149 | | |
| IIV on MAT (%CV) | 56 | 45 - 71 | | |
| IOV on F (%CV) | 71 | 60 - 85 | | |
|  |  |  |  |  |
| **Residuals** |  |  |  |  |
| Proportional error (%CV) | 46 | 41 - 51 | | |

Parameter values reported as means. CL/F, apparent oral clearance; Vc/F, apparent oral volume of central compartment; MAT: mean absorption time; Q/F, apparent oral intercompartment clearance; Vp/F apparent oral volume of peripheral compartment; F, bioavailability; IIV, interindividual variability; IOV, interoccasion variability; 95% CI, 95% confidence interval of parameter values as re-estimated by SIR[8, 9]. Coefficients of Variation (%CV) are calculated according to: %CV=sqrt(exp(ω^2^) – 1) [10]

# Figures

### Supplementary Figure 1: Follow up biochemistry and inflammatory markers

**
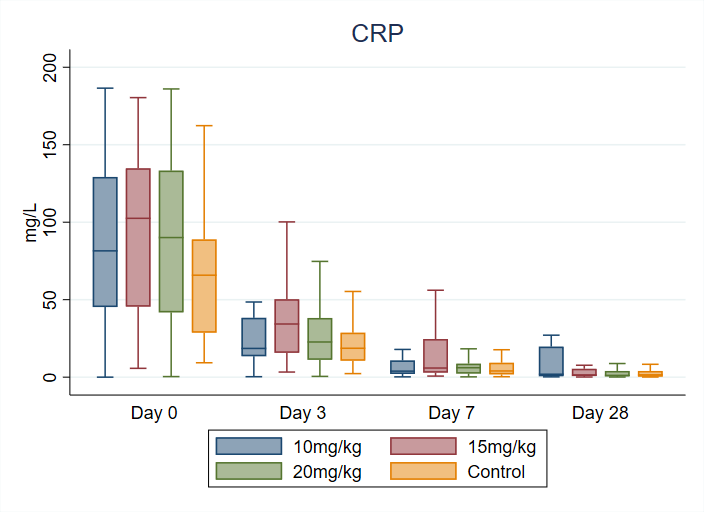
**


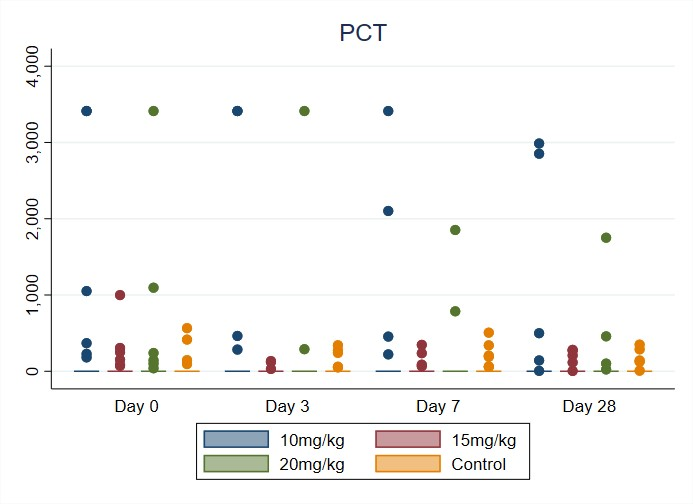
**
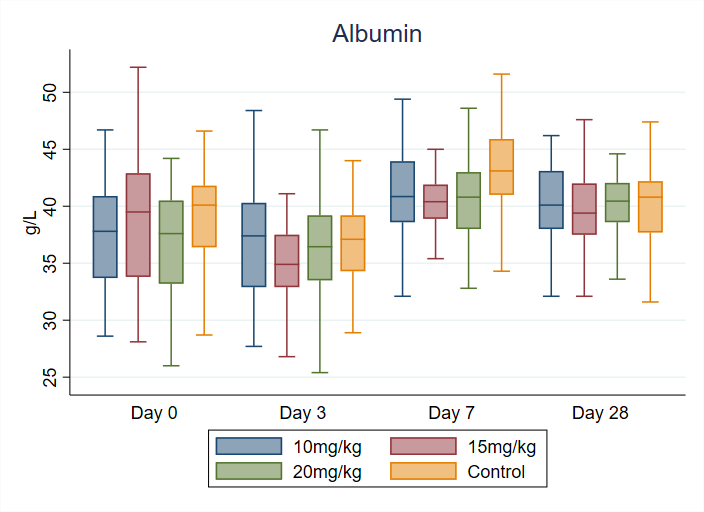

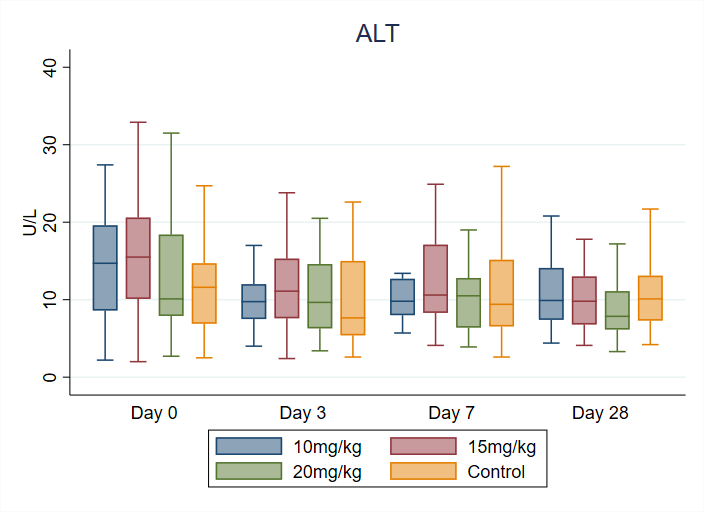

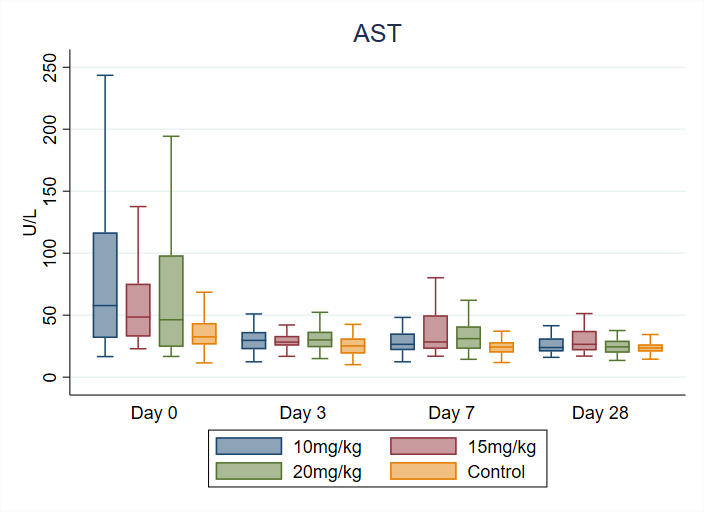

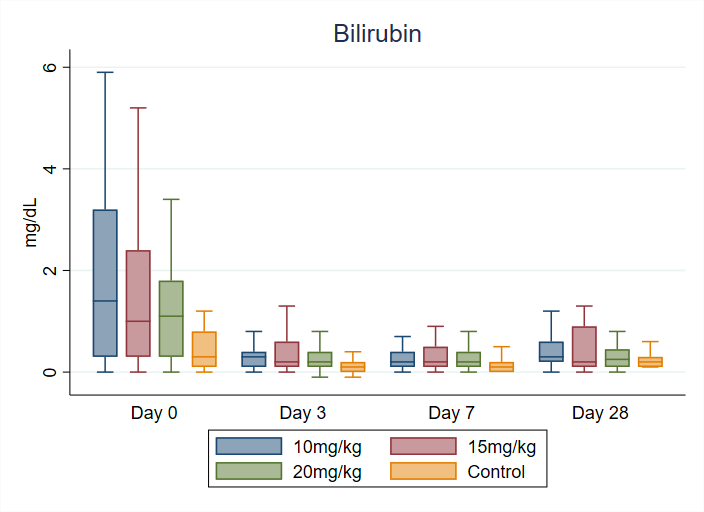

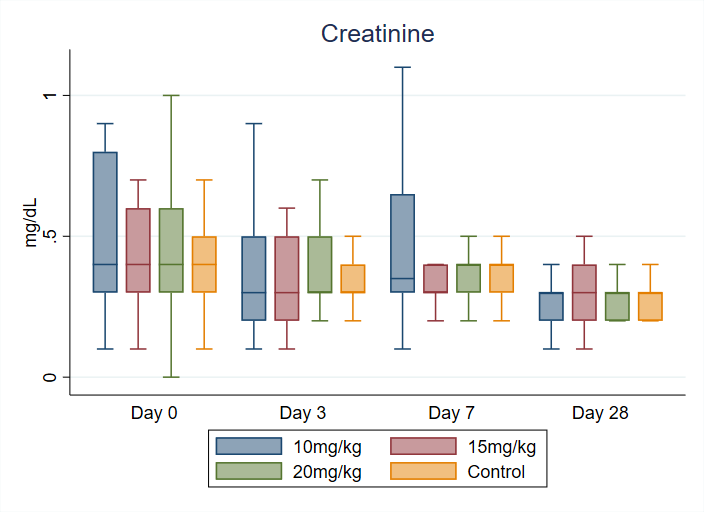
**

**
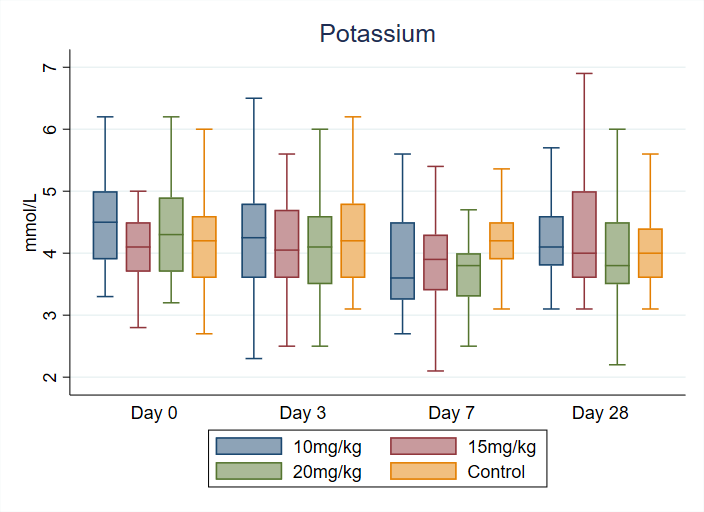
**

### Supplementary Figure 2: Days to discharge

**
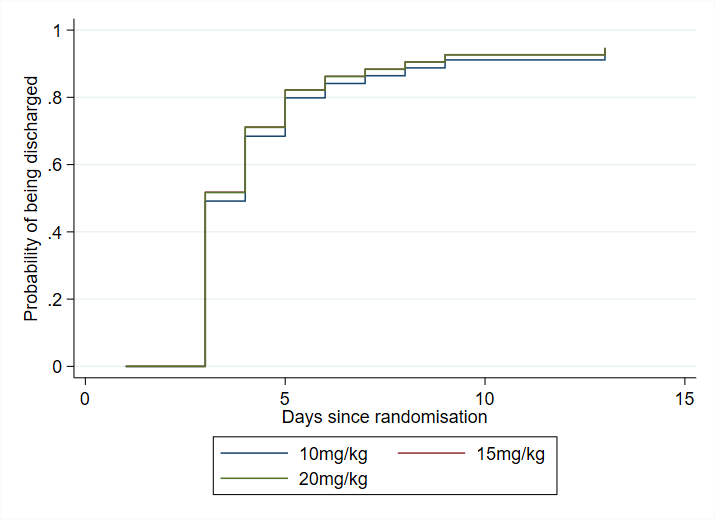
**

### Supplementary Figure 3: Days from discharge to readmission

**
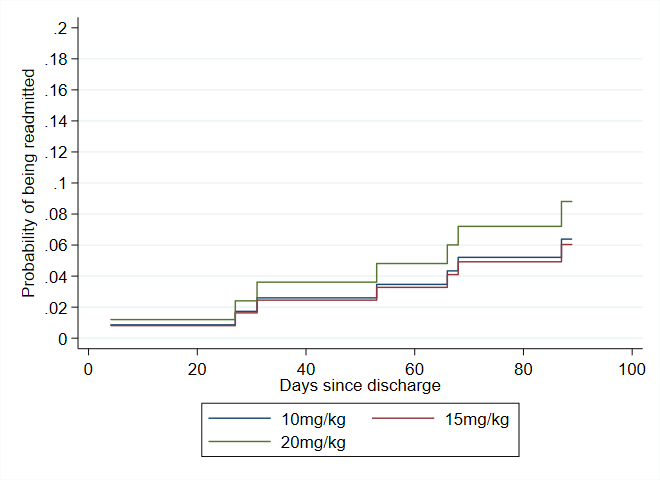
**

### Supplementary Figure 4: Schematic representation of the final PK model


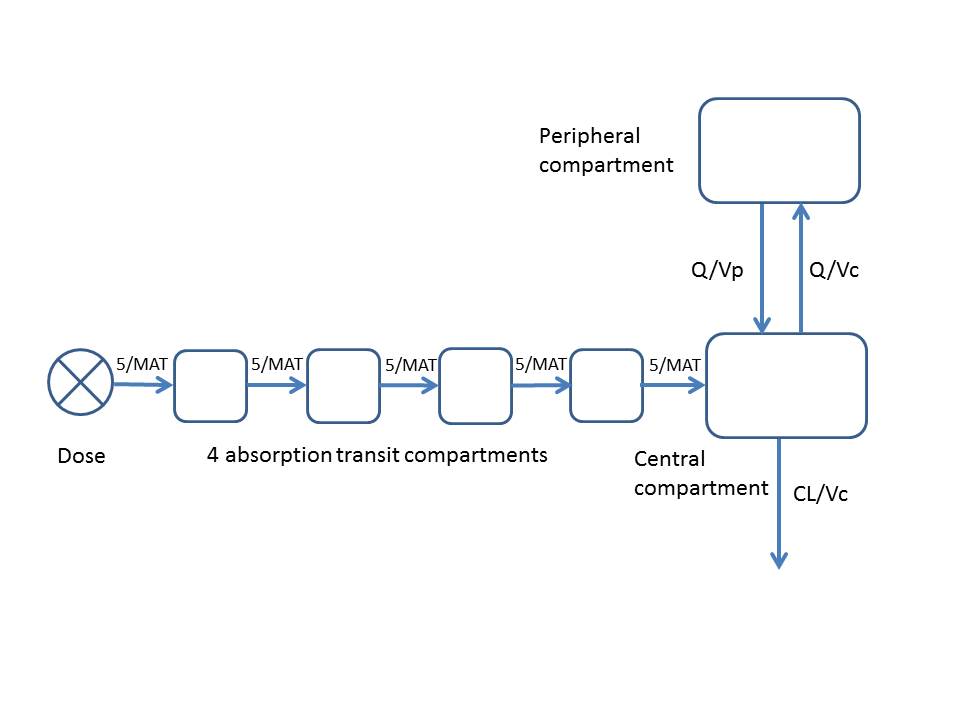


After oral intake of a dose, azithromycin enters the central compartment through a series of four transit compartments[7], reflecting the delayed gastrointestinal absorption process. From the central compartment, the drug is distributed over the peripheral compartment or cleared mainly through biliary excretion. MAT, mean absorption time; CL, clearance; Q, intercompartmental clearance; Vc, volume of central compartment; Vp, volume of peripheral compartment.

### Supplementary Figure 5: Goodness of fit

**
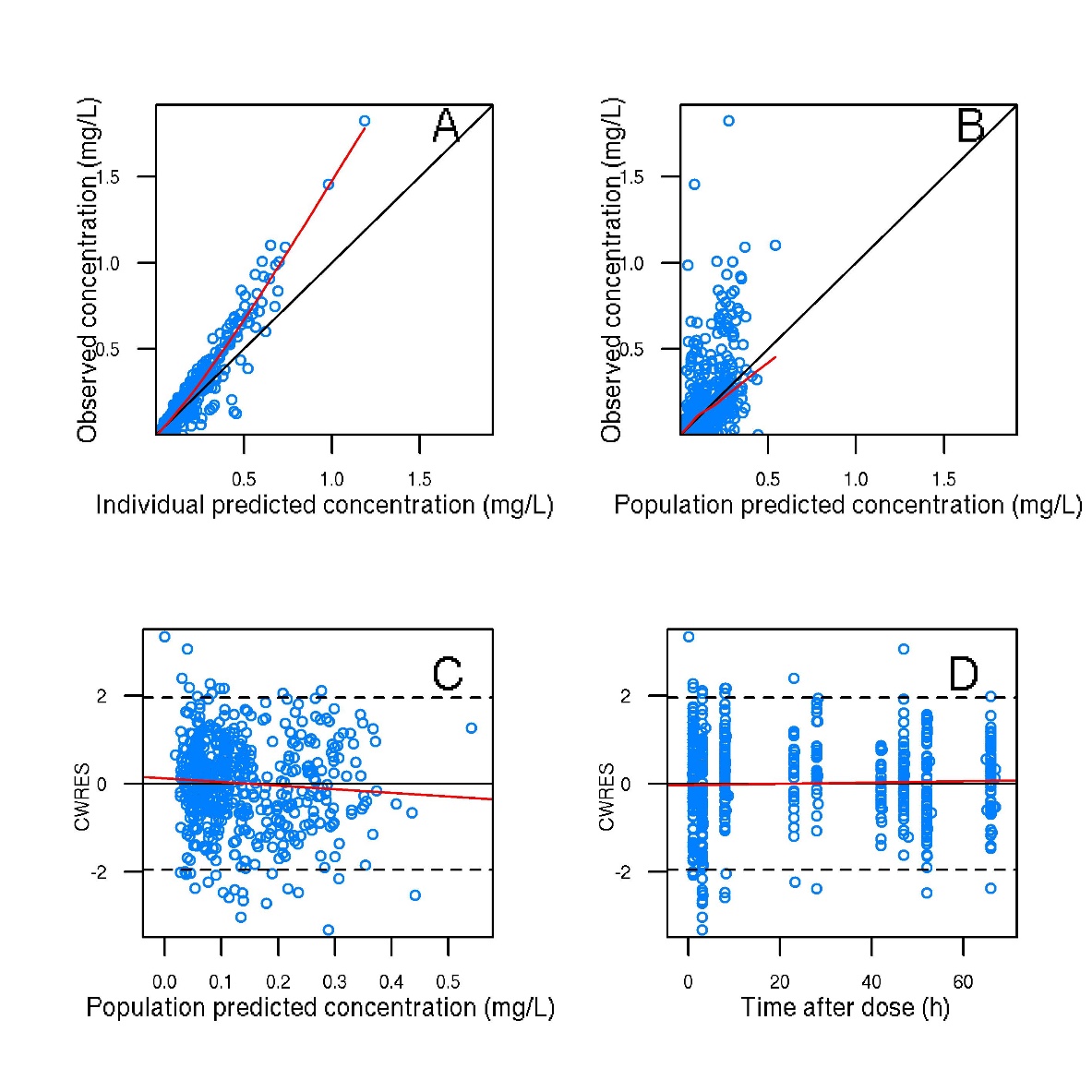
**

Panel A: Observed azithromycin concentration versus predicted individual concentration. The black line indicates identity. The red line is the regression of observed on predicted concentration; Panel B: Observed azithromycin concentration versus population predicted individual concentration; Panel C: Conditional weighted residuals versus population predicted individual concentration; Panel D: Conditional weighted residuals versus time after dose. The residual plots have a flat appearance with only a few data points outside the +/-2 range.

### Supplementary Figure 6: Prediction-corrected visual predictive check


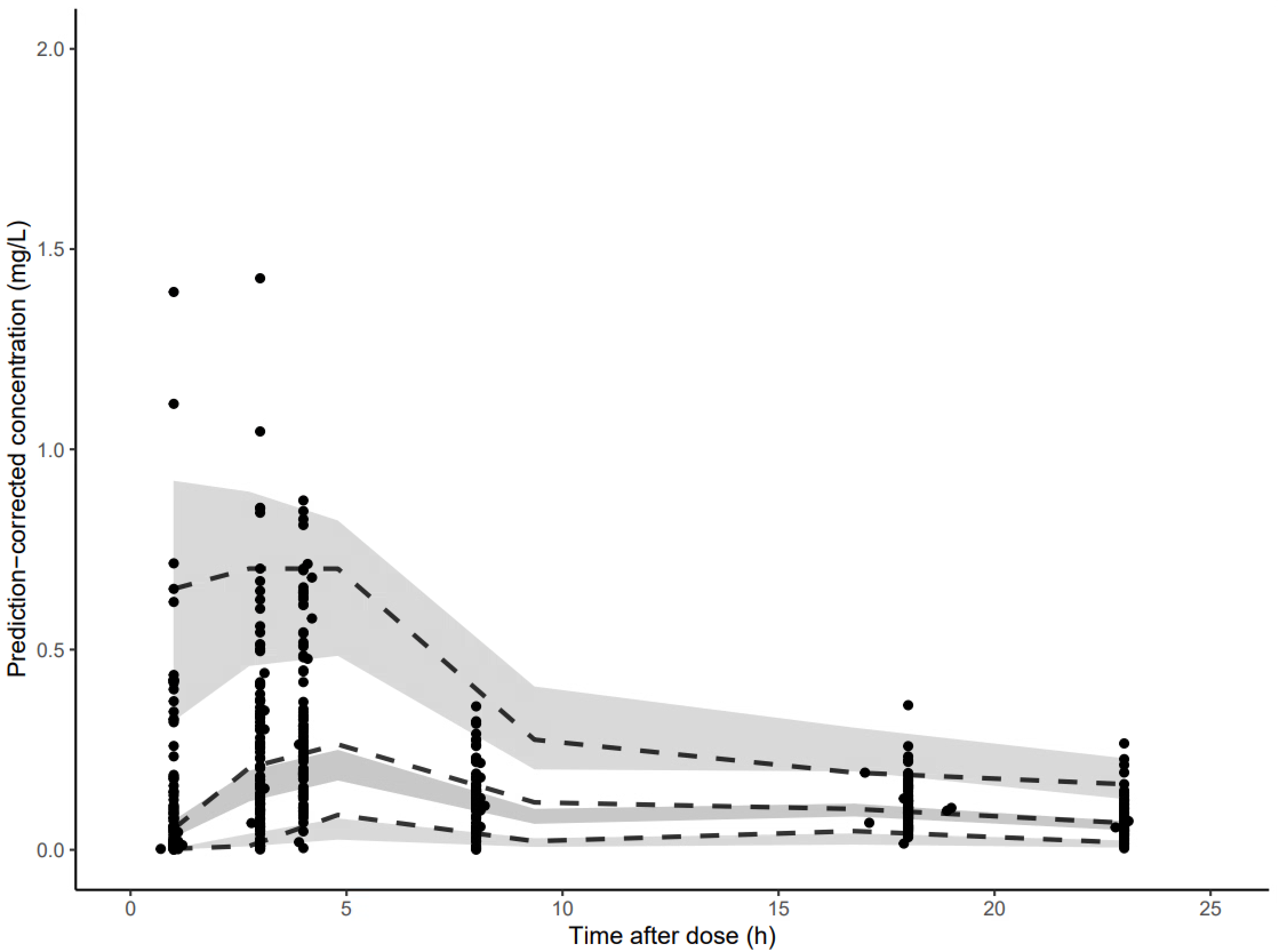


Prediction-corrected visual predictive check for azithromycin in children based on 1000 simulations. The black dots are the individual prediction-corrected observed data points. The dashed lines connect the observed 5th, 50th and 95th percentiles per bin. The grey areas are the 95% confidence interval of the simulated percentiles. The observed percentiles correlate well with the simulated percentiles, visually indicating that the developed model can be used to simulate the observed data.

### Supplementary Figure 7: Reduction in CRP concentration versus azithromycin concentration


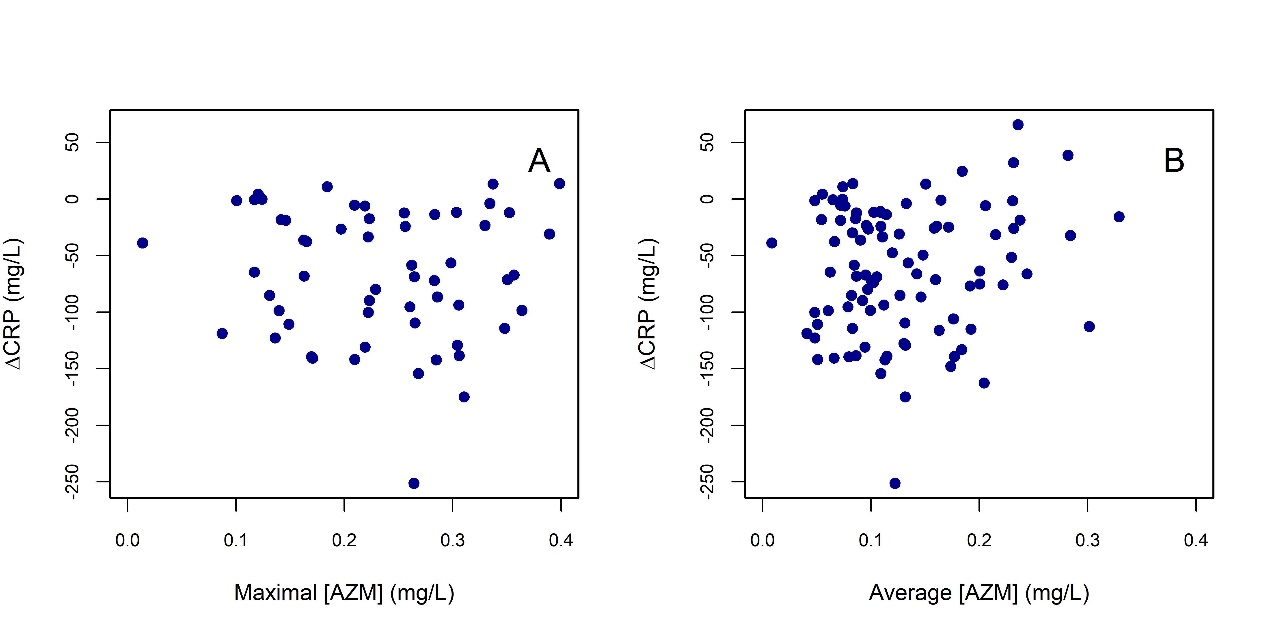


Panel A: Reduction in C-reactive protein concentration versus maximum azithromycin (AZM) concentration after 3 days of treatment. No concentration dependent trend can be observed (Spearman’s rank correlation ρ=0.0227, p-value=0.827); Panel B: Reduction in C-reactive protein concentration versus average azithromycin concentration after 3 days of treatment. No concentration dependent trend can be observed (Spearman’s rank correlation ρ=0.0608, p-value=0.558);

Supplementary Figure 8: Comparison of pharmocokinetic characteristics from conventional and allometric dosing regimens

**
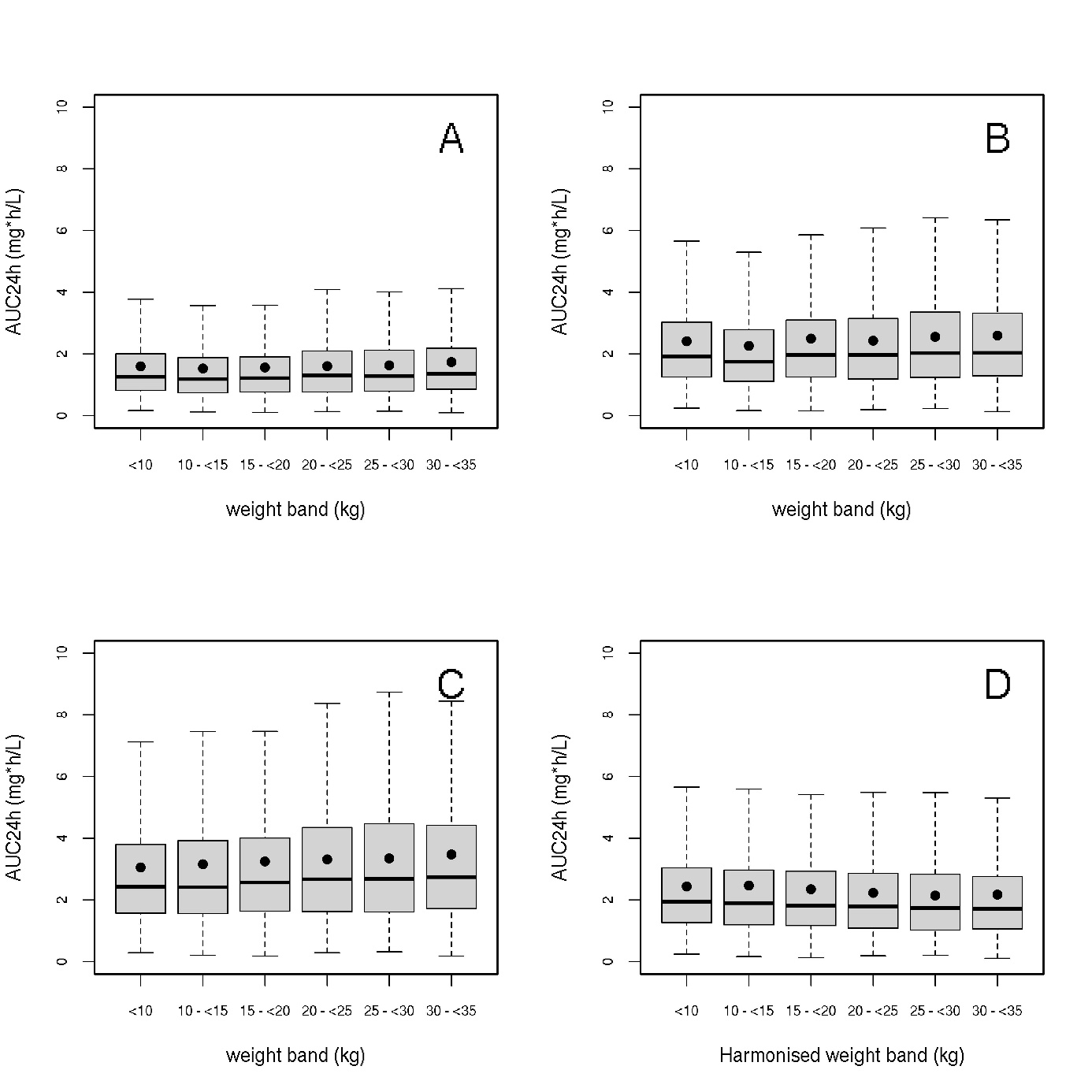
**

Note: Achieved exposure, reported as AUC_0-24h_ on the first day of treatment, using different dosing regimens. The mean reference level in adults is 3.05 mg*h/L on the first day [6]. The solid dots indicate the mean AUC_0-24h_ resulting from the simulation. The boxes represent the predicted interquartile range and the solid black line represents the predicted median. The whiskers depict the extremes. Panel A: 10 mg/kg body weight; Panel B: 15 mg/kg body weight; Panel C: 20 mg/kg body weight; Panel D: Allometric dosing, consisting of a flat dose per weight band according to table 5. This dosing scheme results in a consistent exposure across all weight bands.

### Supplementary Figure 9: NONMEM control stream with final parameter estimates

| $SUBROUTINES ADVAN9 TOL=6  $MODEL  COMP=(DOSE) ;1  COMP=(CENTRAL) ;2  COMP=(PERIPHERAL) ;3  COMP=(TRANS1) ;4  COMP=(TRANS2) ;5  COMP=(TRANS3) ;6  COMP=(TRANS4) ;7  COMP=(AUC) ;8  $ABB COMRES=1  $PK  ; Cmax  IF(NEWIND.LE.1) COM(1)=-1  ; Time after dose needed for creating VPCs  IF (AMT.GT.0) THEN  TDOS=TIME  TAD=0.0  ENDIF  IF (AMT.EQ.0) TAD=TIME-TDOS  ; IOV  IF (OCC.EQ.1) IOV=ETA(4)  IF (OCC.EQ.2) IOV=ETA(5)  IF (OCC.EQ.3) IOV=ETA(6)  IF (OCC.EQ.4) IOV=ETA(7)  ALLOCL=((WT/70)**0.75)  ALLOV=(WT/70)  ALLOK=((WT/70)**(-0.25))  TVCL=THETA(1)*ALLOCL  CL=TVCL*EXP(ETA(1))  TVV2=THETA(2)*ALLOV  V2=TVV2*EXP(ETA(2))  Q=THETA(4)*ALLOCL  V3=THETA(5)*ALLOV  MAT=THETA(3)*EXP(ETA(3))  TVKTR=5/MAT  KTR=TVKTR*ALLOK  ; F1=1; assumed bioavailability  F1=1.00*EXP(IOV) ; assumed bioavailability. parameters represent CL/F, V/F  S2=V2 ; Scaling for observations in central compartment  K14=KTR  K45=KTR  K56=KTR  K67=KTR  K72=KTR  K20=CL/V2  K23=Q/V2  K32=Q/V3  $DES  DADT(1)=-K14*A(1)  DADT(2)=-K23*A(2)-K20*A(2)+K32*A(3)+K72*A(7)  DADT(3)=-K32*A(3)+K23*A(2)  DADT(4)=-K45*A(4)+K14*A(1)  DADT(5)=-K56*A(5)+K45*A(4)  DADT(6)=-K67*A(6)+K56*A(5)  DADT(7)=-K72*A(7)+K67*A(6)  C2=A(2)/V2  IF(C2.GT.COM(1)) THEN  COM(1)=C2  ENDIF  DADT(8)=C2  $THETA  (0, 181) ; CL/F (L/h)  (0, 3140) ; Vc/F (L)  (0, 2.4) ; Mat (h)  (0, 422) ; Q/F (L/h)  (0, 6280) ; Vp/F (L)  $OMEGA  0 FIX ; IIV CL  0.67 ; IIV Vc  0.27 ; IIV Mat  $OMEGA BLOCK(1) 0.41 ; IOV  $OMEGA BLOCK(1) SAME  $OMEGA BLOCK(1) SAME  $OMEGA BLOCK(1) SAME  $SIGMA  0.186 ; PROP ERR  $ERROR  IPRED=F  Y=IPRED*(1+ERR(1))  $EST METHOD=1 INTERACTION NOHABORT MAXEVAL=2000 NSIG=2 SIGL=6  $COV MATRIX=S PRINT=E UNCONDITIONAL |
| --- |

# References

1. **R: A Language and Environment for Statistical Computing**. In*.* Vienna, Austria: R Foundation for Statistical Computing; 2022.

2. Bergstrand M, Hooker AC, Wallin JE, Karlsson MO: **Prediction-corrected visual predictive checks for diagnosing nonlinear mixed-effects models**. *AAPS J* 2011, **13**(2):143-151.

3. Anderson BJ, Holford NH: **Tips and traps analyzing pediatric PK data**. *Paediatr Anaesth* 2011, **21**(3):222-237.

4. Ngai M, Hawkes MT, Erice C, Weckman AM, Wright J, Stefanova V, Opoka RO, Namasopo S, Conroy AL, Kain KC: **Intestinal Injury in Ugandan Children Hospitalized With Malaria**. *J Infect Dis* 2022, **226**(11):2010-2020.

5. Wasmann RE, Svensson EM, Walker AS, Clements MN, Denti P: **Constructing a representative in-silico population for paediatric simulations: Application to HIV-positive African children**. *Br J Clin Pharmacol* 2021, **87**(7):2847-2854.

6. Matzneller P, Krasniqi S, Kinzig M, Sorgel F, Huttner S, Lackner E, Muller M, Zeitlinger M: **Blood, tissue, and intracellular concentrations of azithromycin during and after end of therapy**. *Antimicrob Agents Chemother* 2013, **57**(4):1736-1742.

7. Savic RM, Jonker DM, Kerbusch T, Karlsson MO: **Implementation of a transit compartment model for describing drug absorption in pharmacokinetic studies**. *J Pharmacokinet Pharmacodyn* 2007, **34**(5):711-726.

8. Dosne AG, Bergstrand M, Harling K, Karlsson MO: **Improving the estimation of parameter uncertainty distributions in nonlinear mixed effects models using sampling importance resampling**. *J Pharmacokinet Pharmacodyn* 2016, **43**(6):583-596.

9. Dosne AG, Bergstrand M, Karlsson MO: **An automated sampling importance resampling procedure for estimating parameter uncertainty**. *J Pharmacokinet Pharmacodyn* 2017, **44**(6):509-520.

10. Elassaiss-Schaap JH, S.: **Variability as constant coefficient of variation: Can we right two decades in error?** In: *PAGE meeting.* St. Petersburg; 2009.
